# A common druggable signature of oncogenic c-Myc, mutant KRAS and mutant p53 reveals functional redundancy and competition among oncogenes in cancer

**DOI:** 10.1038/s41419-024-06965-3

**Published:** 2024-08-31

**Authors:** Maria Grześ, Akanksha Jaiswar, Marcin Grochowski, Weronika Wojtyś, Wojciech Kaźmierczak, Tomasz Olesiński, Małgorzata Lenarcik, Magdalena Nowak-Niezgoda, Małgorzata Kołos, Giulia Canarutto, Silvano Piazza, Jacek R. Wiśniewski, Dawid Walerych

**Affiliations:** 1 Mossakowski Medical Research Institute PAS, Warsaw, Poland; 2 Maria Sklodowska-Curie National Research Institute of Oncology, Warsaw, Poland; 3 National Medical Institute of the Ministry of the Interior and Administration, Warsaw, Poland; 4 International Center for Genetic Engineering and Biotechnology, Trieste, Italy; 5 Max Planck Institute of Biochemistry, Martinsried, Germany

**Keywords:** Oncogenes, Proteomics, RNA

## Abstract

The major driver oncogenes *MYC*, mutant *KRAS*, and mutant *TP53* often coexist and cooperate to promote human neoplasia, which results in anticancer therapeutic opportunities within their downstream molecular programs. However, little research has been conducted on whether redundancy and competition among oncogenes affect their programs and ability to drive neoplasia. By CRISPR‒Cas9-mediated downregulation we evaluated the downstream proteomics and transcriptomics programs of *MYC*, mutant *KRAS*, and mutant *TP53* in a panel of cell lines with either one or three of these oncogenes activated, in cancers of the lung, colon and pancreas. Using RNAi screening of the commonly activated molecular programs, we found a signature of three proteins - RUVBL1, HSPA9, and XPO1, which could be efficiently targeted by novel drug combinations in the studied cancer types. Interestingly, the signature was controlled by the oncoproteins in a redundant or competitive manner rather than by cooperation. Each oncoprotein individually upregulated the target genes, while upon oncogene co-expression each target was controlled preferably by a dominant oncoprotein which reduced the influence of the others. This interplay was mediated by redundant routes of target gene activation - as in the case of mutant KRAS signaling to c-Jun/GLI2 transcription factors bypassing c-Myc activation, and by competition - as in the case of mutant p53 and c-Myc competing for binding to target promoters. The global transcriptomics data from the cell lines and patient samples indicate that the redundancy and competition of oncogenic programs are broad phenomena, that may constitute even a majority of the genes dependent on oncoproteins, as shown for mutant p53 in colon and lung cancer cell lines. Nevertheless, we demonstrated that redundant oncogene programs harbor targets for efficient anticancer drug combinations, bypassing the limitations for direct oncoprotein inhibition.

## Introduction

The most deadly cancer types exhibit frequent upregulation of c-Myc and mutations in *KRAS* and *TP53* [1]. However, the understanding of the intersections of molecular programs driven by these universal oncogenes is limited, and has not produced standard therapeutic solutions for cancer treatment [2].

The most frequently altered gene on average in human neoplasia is *TP53*, which encodes the essential tumor suppressor p53. Upon acquisition of missense mutations, TP53 not only loses its suppressive properties but often acquires oncogenic gain-of-function [3, 4]. Nevertheless, none of the drugs targeting mutant p53 directly are yet available as standard therapies [5], many are known for lack of direct efficiency and off-target effects [6].

KRAS missense mutations, which occur at high rates in pancreatic (more than 85% of cases), colorectal (~40%) and non-small cell lung (~30%) cancers, lead to persistent activation of the protein’s GTPase activity and downstream signaling through the PI3K-AKT-mTOR, MAP kinase and RAL pathways, among others [7]. The FDA-approved KRAS inhibitors, sotorasib and adagrasib, targeting the G12C mutation, suffer from emerging resistance in monotherapies [8, 9]. Thus, combinational protocols and inhibitors targeting other KRAS mutants are currently in tests [8, 10].

One of the most commonly upregulated pro-oncogenic factors in cancer is *MYC*, which is the master regulator of a vast transcriptional program crucial for cancer progression [11]. Alterations in *MYC* expression found in various types of cancers make this oncogene an appealing therapeutic target. Despite the use of various strategies to inhibit c-MYC, no clinical therapy is currently available [12].

While the described oncogenes are known to have independent activities, they represent important components of cell transformation, where they functionally interact [2]. KRAS is known to stabilize c-Myc by activating ERK1/2, which phosphorylates c-Myc at serine 62 [13]. Several studies have demonstrated that mutant p53 and MYC may positively influence each other’s levels and activity [14, 15]. Furthermore, the transcriptional signature of mutant p53 in head and neck squamous cell carcinoma was enriched in MYC targets, and mutant p53 augmented c-Myc binding to its target promoters [16]. Analysis of mutant p53 and KRAS interactions was performed in pancreatic ductal adenocarcinoma (PDAC) patients often harboring co-occurring *KRAS* and *TP53* mutations. Kim et al. [17] showed that the KRAS-RAF-MEK-MAPK pathway activates CREB1, which is bound by mutant p53 to upregulate the FOXA1 transcription factor program and PDAC metastasis. Another study revealed that mutant p53 rewires the splicing of GTPase-activating proteins to promote the activation of KRAS signaling [18]. Thus, the interactions described are limited to several mechanisms of cooperation between the trio of the oncogenes.

In this study, to increase the understanding of how interactions between mutant p53, mutant KRAS, and c-Myc affect their downstream programs, we systematically surveyed the transcriptomics and proteomics programs of the oncogenes in a panel of colon, lung, and pancreatic cancer cell lines. We defined common and specific pathways driven by the trio of oncogenes and found that simultaneous targeting of common pathways can be efficient at killing cancer cells of various tissue origins. Interestingly, we discovered that these targets are not upregulated as a result of oncogene cooperation but rather by redundancy and competition, with each target driven dominantly by an oncogene which can be replaced, rather than augmented, by the presence of others. This finding implies that critical molecular pathways controlled by major oncogenes possess a “safety mechanism” that guarantees activation in the context of any of the major oncogenic drivers. The robustness of the oncogene redundancy described here makes these pathways valuable targets for eliminating cancer cells.

## Materials and methods

### Cell lines

RKO (RRID:CVCL_0504), HT29 (RRID:CVCL_A8EZ), PANC-1 (RRID:CVCL_048), MIA PaCa-2 (RRID:CVCL_0428), NCI-H23 (RRID:CVCL_1547), NCI-H1299 (RRID:CVCL_0060), A-549 (RRID:CVCL_0023) DLD-1 (RRID:CVCL_0248), Capan-2 (RRID:CVCL_0026) and BxPC-3 (RRID:CVCL_0186) cell lines were acquired from the American Type Culture Collection (ATCC, Manassas, VA, USA). VMRC-LCD (RRID:CVCL_1787) cell line was purchased from Japanese Collection of Research Bioresources Cell Bank (JCRB, Ibaraki Osaka, Japan). LoVo (RRID:CVCL_0399) cell line was acquired from the European Collection of Authenticated Cell Cultures (ECACC, Salisbury, UK) repository. For all the experiments only low passage numbers (below 15 post acquisition) of cell lines were used. The cells had routinely excluded presence of *Mycoplasma* sp. by qPCR.

A-549, VMRC-LCD, RKO, LoVo, HT29, PANC-1, and MIA PaCa-2 cell lines were cultured in DMEM medium (Gibco, Life Technologies, Rockville, MD, USA) supplemented with 10% fetal bovine serum (FBS, Gibco) and 1% Pen Strep Antibiotics (Gibco). NCI-H23, NCI-H1299, DLD-1, Capan-2, and BxPC-3 cell lines were cultured in RPMI medium (Gibco) supplemented with 10% FBS (Gibco) and 1% Pen Strep Antibiotics (Gibco). Human primary fibroblasts (F02 and F03) were obtained from skin biopsies of healthy subjects based on bioethics committee approval (Nos. 108/2017 and 203/2020) of the Central Clinical Hospital of Ministry of Interior and Administration in Warsaw [19]. Human h-TERT (telomerase reverse transcriptase) immortalized K15 and K21 fibroblasts were a kind gift from Prof. Harm Kampinga (University of Groningen, Netherlands) [20]. All fibroblasts were cultured in DMEM medium (Gibco) supplemented with 10% FBS (Gibco) and 1% Pen Strep Antibiotics (Gibco).

### Lentivirus production and transfection

Lentiviruses for protein overexpression were produced by transient plasmid transfection of HEK293T cells (ATCC). Briefly, HEK293T cells were seeded in 10-cm dishes and the following day were transfected with two second-generation packaging plasmids - 5 µg pMD2.G and 10 µg psPAX2 (gift from Prof. Giannino Del Sal, ICGEB Trieste, Italy) and 15 µg of target lentiviral vectors carrying: Cas9 cds (coding sequence; a gift from Dr Magdalena Winiarska, Warsaw Medical University/MMRC PAS, Warsaw, Poland), c-Myc cds (Addgene #46970, Cambridge, MA, USA), KRAS4B G12V cds (Addgene #35633), p53 R175H or R273H cds (a gift from Dr Maciej Olszewski, IIMCB, Warsaw, Poland). 90 µg of PEI (Sigma Aldrich, Saint Louis, MI, USA) was used for transfection. After 48 h the medium was collected, filtered through 0.45 µm PVDF filters (Merck Millipore, Saint Louis, MI, USA), enriched with 10% of FBS and 1% Polybrene (Sigma Aldrich) and used for target cells infection. After 48–72 h the medium containing viruses was replaced with fresh media with 1 μg/mL Puromycin or 1 μg/mL Hygromycin B Gold (InvivoGen, San Diego, CA, USA), used for selection.

### CRISPR-Cas9-mediated oncogene downregulation

The gRNAs, used for CRISPR-Cas9-mediated *KRAS*, *MYC,* and mutant *TP53* silencing, was annealed from two components: trans-activating crRNA (A35507, Invitrogen, Waltham, MA, USA) and crRNA targeting specific gene (A35509, Invitrogen) or negative control (A35519, Invitrogen), according to manufacturer’s protocol. For *KRAS*, *MYC,* and *TP53* targeting we used a mix of two crRNA in 1:1 proportion (CRISPR577256_CR and CRISPR721685_CR for *KRAS*, CRISPR634316_CR and CRISPR634324_CR for *MYC*, CRISPR718498_CR and CRISPR718512_CR for *TP53*), in order to improve the downregulation efficiency. Cancer cell lines with stable Cas9 overexpression introduced by lentiviral infection were seeded in 6-cm dishes and transfected with gRNA mixes using Lipofectamine MessengerMax (Invitrogen), following manufacturer’s instructions. After 48 h cells were harvested for oncoprotein level validation, RNA-sequencing, and proteomics analysis.

### Western blot and antibodies

The effectiveness of CRISPR-Cas9 treatment or oncogene overexpression/silencing was assessed by western blot. Cells were collected and lysed in NP40 buffer (150 mM NaCl, 1% NP-40, 50 mM Tris-HCl, pH 8.0) with HALT protease inhibitor (Thermo Fisher Scientific, Waltham, MA, USA). After 10 min incubation in Laemmli Sample Buffer in 95 °C, samples were run using 10% SDS-PAGE gel and transferred to nitrocellulose membrane (Merck). Membranes were blocked with 5% fat-free milk in TBS-Tween20 0.1% and incubated overnight with primary antibodies listed in the Supplementary Table 7. Uncropped film photos for blots shown in main figures are provided in supplementary files.

### Proteomics analysis

Cells after CRISPR-Cas9 treatment (in 3 biological replicates of control and each oncogene downregulation) were lysed in buffer (50 mM Tris-HCl, pH 7.8) containing 1% SDS and 0.1 M dithiothreitol and sonicated (Diagenode Bioruptor Plus, Diagenode, Liege, Belgium). Number of sonication cycles was established based on a visual sample clarity assessment. WF-assay was applied in order to calculate protein and peptide concentration [21]. Multi-Enzyme Digestion Filter Aided Sample Preparation (MED FASP) protocol [22] with minor modifications [23] was applied for lysate processing, as described previously [24]. MaxQuanf software (https://maxquant.net/maxquant/) was used for spectra search and total protein approach using the raw protein intensities was applied for protein concentration calculation. Perseus software (https://maxquant.net/perseus/) was used to perform differential analysis, t-tests and assess p-value support of differences between protein concentrations in distinct experimental conditions as well as perform hierarchical clustering. The raw proteomics results are available in Pride database under record number PXD037398.

### RNA-sequencing

Total RNA was extracted from cell lines in 3 biological replicates after each CRISPR/Cas9 oncogene downregulation using QIAzol (Qiagen, Venlo, Netherlands), following the manufacturer’s instructions. Quality and quantity of obtained RNA was analyzed using NanoDrop (Thermo Fisher Scientific) and Experion RNA analyzer (Bio-Rad, San Franscisco, CA, USA). After additional quality control with Qubit (Thermo Fisher) and Bioanalyzer RNA (Agilent, Santa Clara, CA, USA) and libraries preparation (KAPA RNA HyperPrep with RiboErase, HMR), samples sequencing (NovaSeq6000; 100 M reads, 2 × 100 bp) and preliminary data quality check was performed by CeGat GmbH (Tubingen, Germany).

After filtering and trimming of raw data, reads were mapped to the reference human genome, and the abundance of each transcript was calculated (for details see [25]).

Good quality scores were saved in a matrix form and were used as an input for determination of differentially expressed genes (DEG) between control and oncogene downregulation conditions by using DESeq2. The Relative Log Expression method was used in DESeq2 to calculate normalization factors. Benjamini-Hochberg false discovery rate (FDR), FDR < 0.05 was considered as statistically significant parameter of significantly DEG. The count matrix genes were annotated with Ensembl BioMart to extract protein coding genes, which were used to generate principal component analysis plot. Perseus software was used to perform hierarchical clustering of the DEG results.

The raw RNA-seq results are available in GEO database under record number GSE239817.

### Pathway analysis and target gene determination

**Fig. 1:**
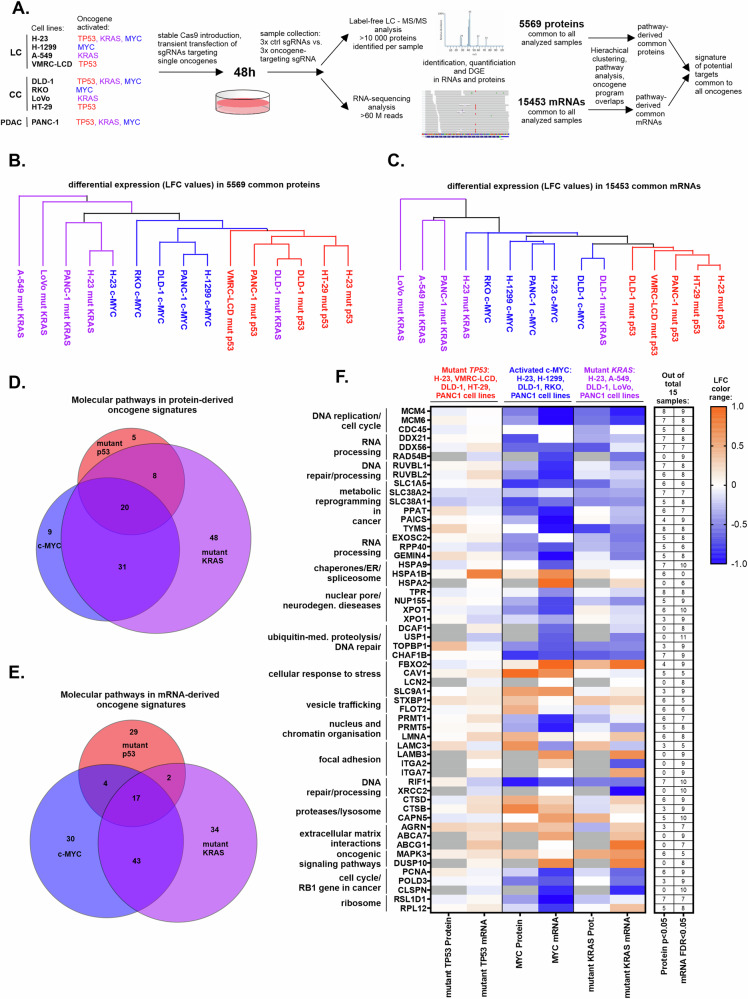
Common targetable pathways driven by mutant p53, mutant KRAS, and c-Myc revealed by proteomics and transcriptomics. **A** Experimental flowchart of the CRISPR-mediated oncogene editing in the indicated cancer cell lines (LC – lung cancer, CC – colon cancer, PDAC – pancreatic ductal adenocarcinoma), followed by differential proteomics, transcriptomic, and their subsequent analyses. **B** Hierarchical clustering (Euclidean distance) of the differentially regulated 5569 proteins common to all the analyzed samples. Colors indicate programs dependent on each of the listed oncogenes. **C** Hierarchical clustering performed as (**B**), for the common 15453 mRNAs differentially regulated in all the samples. **D** Venn diagram showing overlap of pathways significantly (FDR < 0.05) associated by the Clue-GO software with the proteins differentially regulated by each of the three oncogenes indicated by names and colors across all the cell lines with a given oncogenes. The cut-off of *p* < 0.05 for the differential analysis was used for the proteins included in the pathway association, followed by a duplicate filtration in each of the oncogenes’ programs. The significantly (*p* < 0.05) up- and downregulated proteins both were used in the pathway association. **E** Overlap as in (**D**) for mRNA-derived pathways regulated by the indicated oncogenes. **F** A heatmap indicating average protein and mRNA level changes (Log Fold Change – LFC – range indicated at the color scale) of the listed genes derived from the common pathways in (**D**, **E**), in cell lines with mutant *TP53*, mutant *KRAS* or hyperactive *MYC*. The table on the right side shows how many times each of the listed genes was significantly changing level on the mRNA (FDR < 0.05) and protein (*p* < 0.05) levels in all the analyzed samples. The genes are assigned to pathway-derived functional groups shown on the left.

Proteins and mRNAs significantly changing levels (*p* < 0.05 and FDR < 0.05, respectively) in the differential analysis of proteomes and transcriptomes for each oncogene were fused in to signatures and filtered for duplicates. Such signatures were used in ClueGO ver. 2.5.8 (42) plug-in in Cytoscape ver. 3.8.2 (www.cytoscape.org) to associate proteins with molecular pathways. ClueGO settings were: All_Experimental evidence, GO Molecular Pathways/KEGG/WikiPathways ontologies, network specificity slider half way between Medium and Detailed settings, show only pathways with *p* < 0.05. Analyses performed for the separate oncogenes were exported into tables and overlapped (Supplementary Table 3) to determine pathways specific and common to the signatures. Presence of each gene associated with the pathways common to all three oncogenes was then validated in each of the proteomics and transcriptomics significant DEG results from each cell line, and the genes with highest counts were selected to represent the pathways in the heat map in Fig. 1F.

### Gene expression and survival analysis in patient datasets

The analysis was conducted using the patient gene expression data from TCGA datasets for cancer types indicated in the figures, such as lung, colon, pancreatic, stomach, uterine, and bladder urothelial cancer. Patients were stratified based on *MYC* expression levels to “high” and “low”, as below or above mean level in each dataset, and the presence of *TP53* or *KRAS* point mutations. For Fig. 4 a comparative expression analysis of the 3-gene signature (*RUVBL1, HSPA9, XPO1*) was conducted, categorizing patients into specific groups such as *MYC* high only, *KRAS* mutation only, *TP53* missense mutation, then *MYC* high + *TP53* mut + *KRAS* mutation group, and *MYC* low + *TP53* WT/null + *KRAS* WT as a control group. The Student’s t-test was employed to calculate significance for each cancer type separately and box plots were generated using ggplot R packages. For the analysis in Fig. 6, similarity-stratified patient datasets were used to compare expression levels of all mRNA available in each dataset for a given expression/mutation set with the rest of the set. The Student’s t-test with FDR Benjamini-Hohberg correction result cut-off of <0.001 was used to determine mRNAs that significant change is associated with the presence of particular expression/mutation set. Such mRNA lists were used for overlaps shown in ribbon plots in Fig. 6.

Survival analysis in Supplementary Fig. 4 was done using Kaplan-Meier plots with log-rank tests, categorizing patients into low and high expression groups for the 3-gene signature (*RUVBL1, HSPA9, XPO1*). The log-rank test assessed significant differences in survival rates, while hazard ratios estimated relative risks. The findings aimed to elucidate the relationship between the expression levels of signature genes and patients’ survival across diverse cancer types. This analysis was conducted using the Surv function in R, and plots were constructed using the ggplot packages.

### Promoter binding candidates selection

Three genes from the studied signature were each separately input to four online tools assisting determination of transcription factors binding gene promoters: ChEA3 (https://maayanlab.cloud/), EPD The Eukaryotic Promoter Database (https://epd.expasy.org/epd/), CiiiDER (https://ciiider.org/) and ChIP-Atlas (https://chip-atlas.org/). Top hits were overlapped between the genes and the transcription factors suggested by each tool for at least two genes were further considered. The final choice (Fig. 5, Supplementary Table 6) was based on cross-checking the top hits with the literature data confirming functional link with KRAS and/or mutant p53.

### siRNA silencing and mini-screen

For the mini-screen the cells were plated in 96-well plates and transfected with 20 nM of pre-designed siRNAs (purchased from Horizon Discovery – Dharmacon, Lafayette, CO, USA, listed in Supplementary Table 7) with the use of Lipofectamine RNAiMAX (Invitrogen), according to manufacturer’s instructions. 48 h after transfection 1% resazurin (Sigma Aldrich) was added to each well and 2 h later viability was measured.

For experiments with the use of siRNAs in K15 fibrobalsts, cells were seeded in 6–12 well plates, and transfected with indicated siRNAs (listed in Supplementary Table 7) with the use of Lipofectamine RNAiMAX (Invitrogen), according to manufacturer’s instructions. 48 h after transfection cells were harvested and further processed.

### Drug tests

CB-6644, MKT-077 (both purchased from MedChemExpress, Monmouth Junction, NJ, USA) and selinexor (KPT-330, Selleck Chemicals, Houston, TX, USA) were dissolved in DMSO and used in concentrations calculated individually for each cell line, as described in Results section. Cancer cell lines as well as normal fibroblasts were seeded in 96-well plates. After 24 h medium was replaced for fresh one with single drug or drugs combination. 72 h later viability of cells was measured using ATPlite One Step Reagent (PerkinElmer, Waltham, MA, USA).

### Total RNA extraction from cell lines and patient’s samples

Total RNA from cell lines was extracted using RNA Extracol (EURx, Gdansk, Poland), following standard phenol-chloroform RNA isolation protocol. Patient’s frozen samples were homogenized, incubated in RNA Extracol with RNA extraction beads (Diagenode), sonicated using Bioruptor Plus (Diagenode), and further processed according to standard phenol-chloroform RNA isolation protocol. RNA quantity and quality was assessed utilizing NanoDrop spectrophotometer (Thermo Fisher Scientific).

### RT-qPCR

500 ng of total RNA was reverse-transcribed using NG dART RT kit (EURx), according to manufacturer’s protocol. Sensitive RT HS-PCR Mix SYBR (A&A Biotechnology, Gdansk, Poland) reagents were used for qPCR on One Step Plus Real-Time PCR System (Applied Biosystems, Waltham, MA, USA) and CFX Maestro (Bio-Rad). Primers used for qPCR are listed in Supplementary Table 7.

### Human frozen tissue samples

Sample collection and further laboratory experimental procedures were performed based on ethical committee approvals: No. 109/2016 (with updates) of National Medical Institute of the Ministry of the Interior and Administration in Warsaw, Poland and No. 55/2023 of National Institute of Oncology in Warsaw, Poland. Written consents for research use of collected tissues were obtained from all patients. Altogether 28 samples of colon cancer and 18 samples of pancreatic cancer were collected from patients undergoing surgical treatments (Supplementary Fig. 4). Samples, after surgical resection, were subjected to preliminary histopathological assessment and further storage in culturing medium at 4 °C for organoid culture establishing or in liquid nitrogen for further DNA, RNA and protein extraction. For tumor type and grade classification according to WHO guidelines, tissues were fixed in 10% buffered formalin, embedded in paraffin, microtome cut into 4 mm thick sections and HE (haemotoxylin/eosin) stained.

### Human colon and pancreatic cancer organoid cultures

Colon cancer and normal colon tissues were transported at 4 °C in culturing medium w/o growth factors and processed within 18 h from resections. Protocol from [26] with small modifications was used to generate colon organoids. Details are described elsewhere [25]. Pancreatic organoids were generated in a similar way to colon organoids based on [27], with addition of human Gastrin I 10 nM (Tocris, Bristol, UK) and no SB202190 in the culturing medium. Prostaglandin E2 10 nM (Tocris) was added only to medium for normal tissue organoids.

### Drug sensitivity assays in organoids

33 μL of Matrigel or Basement Membrane Extract type 2 were added to each well of 96-well plate. Plates were then centrifuged at 1000 RCF for 1 min and placed in a 37 °C, 5% CO_2_ incubator for 30 min. To each well a 100 µl suspension of approximately 500 organoids in the culturing medium were added. After 24 h the drugs were added in the culturing medium at concentrations indicated in the figures. The organoids were incubated with drugs for 72 h, then cell viability was measured using ATPlite One Step Reagent (PerkinElmer).

### Chromatin immunoprecipitation

ChiP was carried out essentially as described in [23]. Promoter occupancy was calculated using Fold Enrichment Method (2 − ΔΔCt method). PCR primers for expected c-Myc biding sites were designed using ChIP-Atlas (https://chip-atlas.org/) and are listed in the Supplementary Table 7.

### Statistical analysis

GraphPad Prism 8.0.2 was used for statistical analysis and data visualization. Data are presented as mean ± standard deviation (SD) or standard error of the mean (SEM). Number of replicates as well as applied statistical test are described in each figure legend.

## Results

### Proteomics and transcriptomics reveal common, targetable pathways driven by mutant p53, mutant KRAS, and c-Myc

To reveal protein and RNA populations controlled by oncogenes – mutant *TP53*, mutant *KRAS*, and *MYC* – we used CRISPR-Cas9-mediated editing to downregulate their expression levels in a panel of cell lines derived from cancer types frequently driven by the mentioned oncogenes (Fig. 1A). We stably introduced Cas9 to cell lines of lung and colon cancer which either harbor co-expressed missense hotspot mutant *TP53*, *KRAS*, and oncogenic *MYC* or each of the oncogenes individually. Additionally we performed the same procedure in a pancreatic cancer cell line PANC1, which, as in the majority of PDACs, contains coexpressed *KRAS* and *TP53* mutants, and hyperactive *MYC* (Fig. 1A). Pairs of gRNAs have been introduced by transient transfection to cell lines with stably overexpressed Cas9 to perform NHEJ-mediated knockouts of the oncogenes. Samples were collected and controlled for oncoprotein levels at 48 h post transfection (Supplementary Fig. 1A) to avoid molecular program compensation in cells cultured for longer time periods without one of the main driver oncogenes. The results of a protein and mRNA differential analysis between control and oncogene targeting-sgRNA samples (Supplementary Tables 1, 2, Supplementary Fig. 1B, C), were included in the hierarchical clustering (5569 proteins and 15453 mRNA with quantified differences across all the samples). This revealed that the change values clustered preferentially with each of the oncogenes, rather than the tissue of origin or the cell line (Fig. 1B, C). Hence, we proceeded to evaluate the downstream functionality of these molecular programs in the oncogene-focused manner.

We fused the lists of proteins and mRNAs differentially regulated by each oncogene in all the cell lines, with a cut-off of *p* < 0.05 for proteins and FDR < 0.05 for mRNAs, filtered the resulting lists for duplicates, and performed pathway analysis on each oncogene signature. The molecular pathways were subsequently overlapped to understand specific and common aspects of the oncogene-driven programs (Fig. 1D, E; Supplementary Table 3). The analysis revealed a significant number of common pathways, and we further focused on the common programs of the oncogenes to search for potential universal downstream targets in cancer—both processes and individual proteins/genes. To extract these potential targets from the common pathways we scored how many times each protein or gene from the common pathways was present as a significant (p/FDR < 0.05) change in expression in each cell line. The protein/mRNA level change heatmap for the most frequently regulated proteins and mRNAs driven by the trio of the oncogenes is shown in Fig. 1F. The heatmap shows average changes in mRNA/protein levels (intensity) and change direction (color) in cell lines sharing each oncogene – demonstrating that mutant *TP53* is on average the least strong regulator of the target gene protein and mRNA change, compared to mutant *KRAS* and hyperactive *MYC*. The genes have been assigned to functional groups (Fig. 1F) derived from common oncogene driven processes (Supplementary Table 3). These groups were targeted in an siRNA mini-screen to find specific vulnerabilities in the common molecular programs driven by mutant *TP53*, mutant *KRAS*, and hyperactive *MYC*, as described in the next section.

### Targeting the signature controlled by mutant p53, mutant KRAS, and overexpressed c-Myc is efficient at killing lung, colon and pancreatic cancer cells

**Fig. 2:**
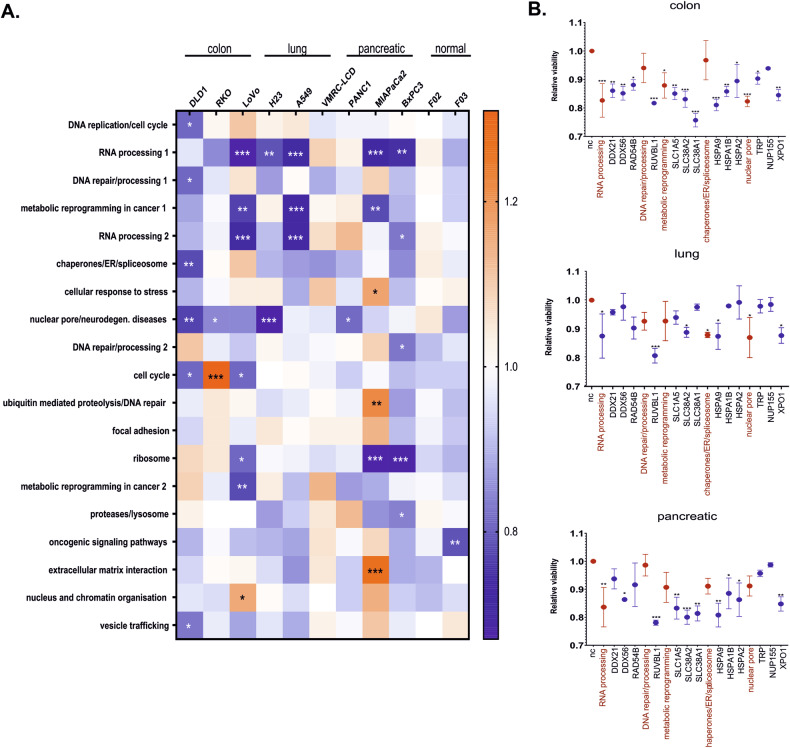
Identification of XPO1, HSPA9, and RUVBL1 as druggable signature controlled by c-Myc, mutant KRAS, and mutant p53. **A** Colon, lung, and pancreatic cancer cell lines and two normal fibroblast lines were transfected with a mixture of 2–4 siRNAs targeting genes belonging to functional groups (siRNAs listed in Supplementary Table 4). A resazurin assay was used to measure cell viability 48 h post transfection. The data in the heatmap are presented as the means of *n* = 2 biological replicates for each cell line and were analyzed with two-way ANOVA (uncorrected Fisher’s LSD) versus the siRNA negative control. **B** Viabilities of colon (DLD1, RKO, LoVo), lung (H23, A549, VMRC-LCD) and pancreatic (PANC1, MIAPaCa2, BxPC3) cancer cell lines treated with a single (dark blue) or combination of siRNAs (red) best performing in the siRNA mini-screen (**A**), targeting helicase activity, ATPase activity, amino acid transport, chaperones and the nuclear pore complex/transport. Viability was measured as described in (**A**). Each result is presented as the mean of *n* = 6 (two biological replicates for each cell line), and the error bars represent the SEM. The data were analyzed with one-way ANOVA (uncorrected Fisher’s LSD). **A**, **B**: *p < 0.05, **p < 0.01, ***p < 0.001.

Overall, nineteen pathways, indicated in bioinformatics analysis as common to molecular programs controlled by mutant *TP53*, mutant *KRAS*, and overexpressed *MYC*, were targeted with sets of two to four siRNAs (Fig. 2A, Supplementary Table 4). The siRNA mini-screen was performed in a panel of nine cell lines of colon (DLD1, LoVo, RKO), lung (H23, A549, VMRC-LCD), and pancreatic (PANC1, MIAPaCa2, BxPC3) cancers, as well as in two untransformed, normal human fibroblasts as control cells (F02, F03), with the aim of identifying functional gene groups whose depletion decreased the viability of cancer cell lines but not that of normal cells.

Targeting helicases (DDX21, DDX56, RAD54B), amino acid transporters (SLC1A5, SLC38A2, SLC38A1), and nuclear pore complex/transport components (TPR, NUP155, XPO1) decreased cancer cell line viability most significantly compared to that of normal cells, while we also observed a tendency to decrease viability across the tested cell lines in the case of blocking DNA repair/processing activity (RUVBL1, RUVBL2) and targeting chaperones (HSPA9, HSPA1B, HSPA2). Subsequently we tested the siRNAs from selected groups individually in order to determine depletion of which individual protein had the most significant impact on cancer cells survival (Fig. 2B, Supplementary Table 4). The results suggested that siRNAs targeting *RUVBL1*, *HSPA9, XPO1*, and *SLC38A2* resulted in the strongest decrease in cancer cell viability but not of normal fibroblasts (Supplementary Fig. 2D); thus, we concluded that the proteins encoded by these genes could lie at the intersection of vital programs of cancers driven by mutant *TP53*, mutant *KRAS*, and hyperactive *MYC* and could be tested as therapeutic targets. Moreover, silencing of the oncogenes directly resulted in a less significant decrease in the viability of the same cancer cell line panel (Supplementary Fig. 2A, B).

**Fig. 3:**
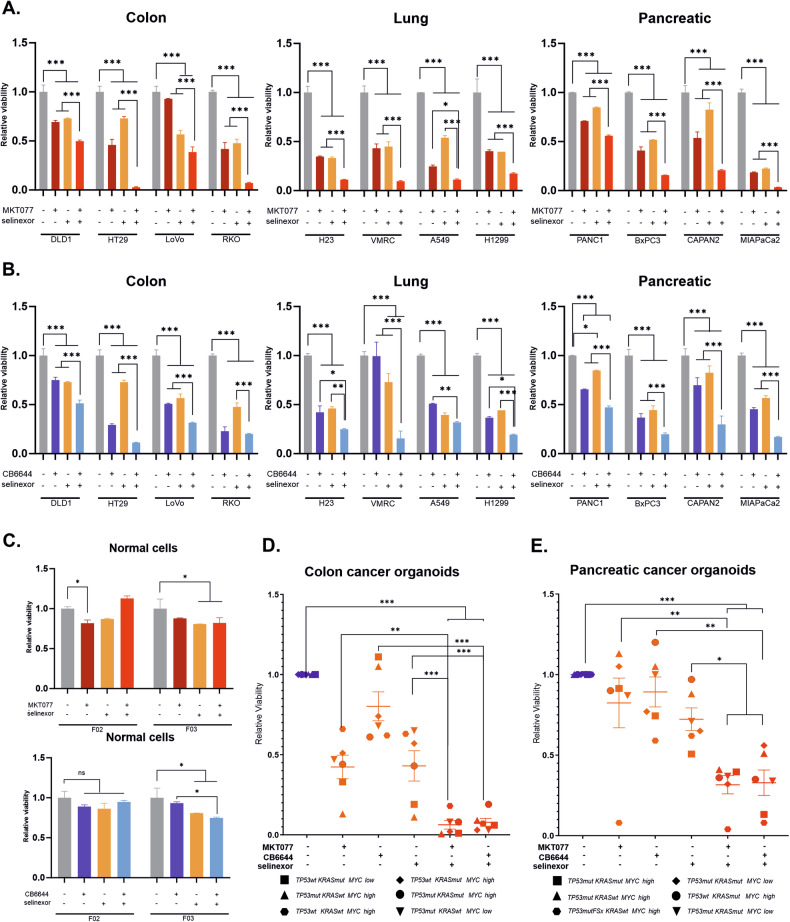
Targeting XPO1 with HSPA9 and XPO1 with RUVBL1/2 efficiently kills cancer cell lines and patient-derived organoids. **A** Impact of MKT077 (an HSPA9 inhibitor) and selinexor (a nuclear exportin 1 inhibitor), used as single agents and in combination, on the viability of the indicated colon, lung and pancreatic cancer cell lines. **B** Viability of the listed colon, lung and pancreatic cancer cell lines treated with CB6644 (an inhibitor of RUVBL1/2 ATPase activity), selinexor or a combination of both inhibitors. **C** Viability of normal fibroblasts upon treatment with the indicated combinations of MKT077, selinexor and CB6644. Viability (**A**–**C**) was measured with ATPlite at 72 h post treatment, and the drug concentrations were calculated for each cell line (based on the IC_50_ values). Each bar represents the mean of two replicates with the SD. The data were analyzed via two-way ANOVA with Tukey’s correction; *p < 0.05, ***p < 0.01. **D**, **E**. Viability of 6 colon and 6 pancreatic organoids derived from tumor patient tissue harboring the listed mutations in *TP53* and *KRAS* and high/low c-Myc levels. MKT077 (5 µM), CB6644 (2 µM) and selinexor (2 µM) were used in combination as indicated in the graphs. Viability was measured 72 h after treatment using an ATPlite assay. One-way ANOVA with Sidak’s correction was applied, *p < 0.05, **p < 0.01, ***p < 0.001.

Subsequently we targeted proteins encoded by genes revealed in siRNA mini-screen as potential vulnerabilities common to mutant *TP53*, mutant *KRAS*, and hyperactive *MYC* programs. We tested the effect of CB6644 (targeting RUVBL1/2 complex), MKT077 (inhibiting HSPA9), selinexor (blocking XPO1, nuclear exportin), and MeAIB (targeting SLC38A2) on cell lines representing three different cancer types versus the normal fibroblasts. Each cancer cell line was treated with selected inhibitors used as single agents or in combination, with concentrations corresponding to the obtained IC_50_ values (Supplementary Fig. 2F, Supplementary Table 5). MeAIB inhibitor did not affect significantly the viability of cancer cell lines, thus we decided to exclude this inhibitor from further experiments (data not shown). A significant decrease in viability was observed in simultaneous XPO1 + HSPA9 or RUVBL1/2 targeting (Fig. 3A, B) in comparison with normal fibroblasts (Fig. 3C) and typical chemotherapy protocols used for colon, lung or pancreatic cancers (Supplementary Fig. 3C). K21 immortalized fibroblasts with introduced trio of the oncogenes (single or in pairs) were more sensitive to inhibitors than control with no oncogenes introduced, further supporting the hypothesis, that oncogene-driven cells are more sensitive to our inhibitor combinations than untransformed cells (Supplementary Fig. 2E). Combination of MKT077 and CB6644, as well as the use of triple mixture of inhibitors, were less effective in decreasing cancer cell lines viability (Supplementary Fig. 3A, B). However, it needs to be mentioned that the therapeutic effect of the inhibitors mixture may, in part, be due to their off-target effects (Supplementary Fig. 2C).

To further confirm these findings we used an in vitro model of heterogeneous, patient-derived organoid cultures of pancreatic and colon cancers. The combination of selinexor with either MKT077 or CB6644 efficiently killed organoid cultures harboring mutations in *TP53*, and/or *KRAS*, and/or high c-Myc levels (Fig. 3D, E; Supplementary Figs. 3D, E, and 4H, J). Obtained results altogether suggested that the combined blockade of exportin 1 and RUVBL1/2 complex, as well as inhibiting exportin 1 and HSPA9, are promising targeted therapeutic approaches at least against cancers driven by mutant *TP53*, mutant *KRAS*, and/or hyperactive *MYC*.

### The druggable signature expression is associated with the presence of any of the activated trio of oncogenes in patient samples

**Fig. 4:**
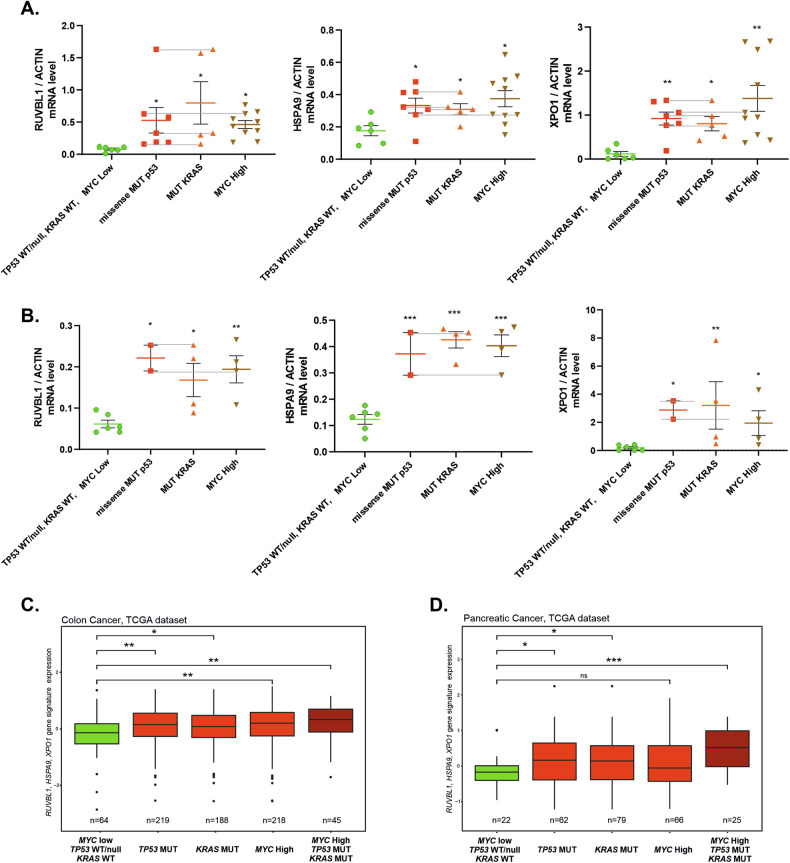
Dependence of RUVBL1, HSPA9, and XPO1 expression on oncogenic c-Myc, mutant KRAS, and mutant p53 in patient-derived cancer samples. **A** The expression of the *RUVBL1, HSPA9*, and *XPO1* genes was tested in 24 colon cancer samples stratified according to c-Myc expression (samples above average *MYC* expression for all samples are considered “High”) and the presence of missense *TP53* and *KRAS* mutations. The samples with two coexisting oncogene activation conditions are linked with horizontal lines. **B** The same expression analysis as in (**A**) was used for 14 pancreatic cancer samples. One-way ANOVA with Dunnett correction *p < 0.05, **p < 0.01, ***p < 0.001. **C** Comparative expression analysis of a 3-gene signature consisting of *RUVBL1, HSPA9*, and *XPO1* in TCGA-derived colon cancer patient samples (the mean value of three genes in each patient was used to calculate the sample distribution in the box plot), stratified according to the listed *TP53, KRAS* (point mutations only), and *MYC* expression status. The sample was included in the “c-Myc high” group if the *MYC* gene expression was above the *MYC* average expression level for all the patients in the graph. Student’s t-test was used to analyze the differences, *p < 0.05, **p < 0.01, ***p < 0.001. The same procedure was used for (**D**) for TCGA-derived patient samples of pancreatic cancer.

To verify whether the expression of the signature of the 3 genes - *RUVBL1*, *HSPA9*, and *XPO1 -* which encode the proteins targeted in Fig. 3 is indeed associated with the presence of mutant p53, mutant KRAS, or high levels of c-Myc, we interrogated cancer patient samples. We tested the relative expression of the trio of individual genes in 24 locally collected samples of colon cancer (Fig. 4A) and 14 from pancreatic cancer (Fig. 4B). We stratified the samples according to their *MYC* expression levels (“high” was above the mean of all samples for each cancer type) and the presence of *TP53* or *KRAS* hotspot mutations (Supplementary Fig. 4G, I). In case of each of the 3 tested genes, the level of expression was, on average, significantly higher in samples containing any of the mutations and/or high *MYC* expression (Fig. 4A, B), than the group of samples which contained none of the activated oncogenes (Fig. 4A, B). These findings indicated that high expression of *RUVBL1*, *HSPA9*, and *XPO1* was significantly associated with the presence of any of the trio of activated oncogenes in the patient samples of colon and pancreatic cancers.

To test if this association occurred in larger populations of patients in publically available datasets, we tested the signature consisting of *RUVBL1*, *HSPA9*, and *XPO1* genes against those of TCGA-derived cancer patient datasets which contained sufficient *TP53* and *KRAS* mutations for statistically relevant analyses (see Materials and Methods). In all the analyzed cancer types, the presence of at least one activated oncogene of the studied *KRAS/TP53/MYC* trio was sufficient to significantly increase the expression of the signature (*p* < 0.05) above the control group of patients (*MYC* expression below the average for all samples, and the WT *KRAS/TP53* status). In all cancer cases, the co-presence of all three activated oncogenes was not resulting in the signature expression significantly higher than with a single active oncogene (Fig. 4C, D; Supplementary Fig. 4A–D). We obtained a similar result also in the case of broad, 40-gene signature, comprised of all on-average oncogene-upregulated transcripts from Fig. 1F (Supplementary Fig. 4F). This suggested that the oncogene activation is redundant rather than cooperative in increasing the signature expression.

### The expression of the targetable signature is redundantly and competitively controlled by the activated oncogenes

**Fig. 5:**
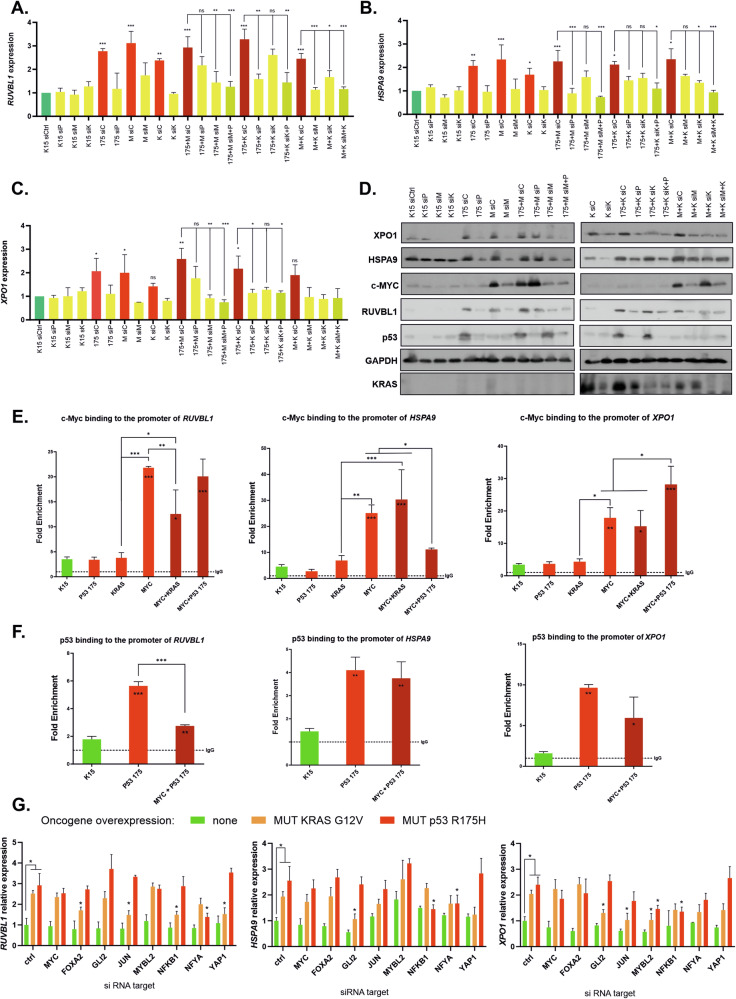
Mechanism of RUVBL1, HSPA9, and XPO1 expression control by oncogenic c-Myc, mutant KRAS, and mutant p53. **A**–**C** Relative mRNA expression levels of the *RUVBL1, HSPA9*, and *XPO1* genes (respectively) in K15 immortalized human fibroblasts upon the indicated stable lentiviral vector-mediated overexpression or transient (48 h) silencing of the oncogenes (overexpression: P – mutant p53 R175H, K – mutant KRAS, M – wt c-Myc; siX – siRNA-mediated silencing of the oncogene indicated by the letter X). **D** Western blot of the indicated proteins in the K15 immortalized human fibroblasts upon oncogene overexpression or silencing performed as described in (**A**–**C**). **E** Chromatin immunoprecipitation-derived qPCR results of the predicted c-Myc-binding regions using anti-c-Myc antibodies in the promoters of the indicated genes performed in K15 immortalized human fibroblasts with stable oncogene overexpression or co-overexpression. The PCR results were normalized to the level of IgG nonspecific antibody background controls used for ChIP in parallel to specific antibodies in each oncogene overexpression setup. **F** Chromatin immunoprecipitation-derived qPCR results of the predicted c-Myc-binding regions performed and normalized as described in (**F**), but using anti-p53 antibodies. **G** Relative mRNA expression levels of the *RUVBL1, HSPA9*, and *XPO1* genes in K15 immortalized human fibroblasts upon the indicated stable lentiviral vector-mediated overexpression of mutant *TP53* or *KRAS* and transient (48 h) silencing of the listed candidate transcription cofactors. **A**–**G** The means with SDs are shown for 2–3 biological replicates (for each mean of 2 technical replicates). One-way ANOVA with Dunnett correction *p < 0.05, **p < 0.01, ***p < 0.001.

We proceeded to assess whether the activated oncogenes *MYC*, mutant *KRAS*, and *TP53* control the expression of the *RUVBL1, HSPA9*, and *XPO1* genes in a cooperative, redundant, or competitive manner. We used a uniform cellular background of K15 fibroblasts immortalized by the introduction of hTERT [20] to overexpress and silence each of the oncogenes individually or in pairs (Supplementary Fig. 5A). The following observations were made in this experiment (Fig. 5A–C for mRNA levels and Fig. 5D for protein levels): (i) each of the oncogenes - *MYC*, mutant *KRAS*, and mutant *TP53* – was individually able to activate each of the three target genes when compared to the expression level in the control K15 fibroblasts; (ii) there was no significant increase in the expression of the targets when the oncogenes were co-overexpressed in pairs compared to overexpression of the individual oncogenes; (iii) the expression of the targets decreased to the control levels upon silencing of the individually overexpressed oncogenes; and (iv) when the oncogenes were overexpressed in pairs in most cases the silencing of only one oncogene caused a significant decrease in target gene expression. Interestingly, this last effect did not include the same oncogenes for all the targets (Fig. 5A–C). All these results suggested that the expression of the analyzed oncogene targets is redundantly rather than cooperatively controlled by all the studied oncogenes. The co-presence of the oncogenes usually led to a dominance of one of them in inducing the target gene’s expression, which implied the presence of oncogene competition.

To investigate whether competition between the oncogenes for dominance in controlling target gene expression is reflected in the binding of transcription factors to target gene promoters, we performed chromatin immunoprecipitation (ChIP, Fig. 5E, F). Both c-Myc and mutant p53 are known to bind to promoters (directly and indirectly, respectively), and to cooperate in this process [14, 16]. We assessed their binding to the promoter regions of *RUVBL1*, *HSPA9*, and *XPO1* with the predicted c-Myc binding sites in K15 immortalized fibroblasts with introduced oncogenes. We observed a significant increase in the binding of c-Myc and the R175H mutant p53 to all the promoters upon overexpression in K15 fibroblasts. In all cases, except the *XPO1* promoter, there was either no effect of one oncogene on another, or we observed a significant decrease in the binding of one oncogene in the presence of the other (Fig. 5F). This result was consistent with earlier observations that when oncogenes were co-present, c-Myc dominated the control of *RUVBL1* and *XPO1* expression, while mutant p53 dominated over c-Myc in the case of *HSPA9* expression (Fig. 5A–C). All these results suggested independence or competition between the oncoproteins, which eventually led to redundant activation of the studied target genes.

The ChIP experiments (Fig. 5E, F) suggested that mutant KRAS and mutant p53 may regulate the expression of the analyzed targets independently of c-Myc. To identify the mediators of this activation we analyzed the promoters of the *RUVBL1*, *HSPA9*, and *XPO1* genes using multiple online tools, to find candidate transcription factors which may be induced by KRAS and mutant p53 (Materials and Methods, Supplementary Table 6). Silencing of these selected transcription factors in mutant *KRAS-* and mutant *TP53*-overexpressing K15 fibroblasts (Supplementary Fig. 5B) led to the identification of significant dependencies, including GLI2 and c-Jun which regulated expression of two genes each in the presence of overexpressed KRAS, while NFKB1 and NFYA (known to cooperate with mutant p53 [2]) regulated expression of two genes each in the presence of overexpressed mutant p53 (Fig. 5G). We additionally confirmed that GLI2 and c-Jun indeed bind the *RUVBL1*, *HSPA9*, and *XPO1* promoters in the presence of the overexpressed KRAS mutant (Supplementary Fig. 5C).

These results indicate that each of the trio of the studied oncogenes has independent signaling routes that lead to the activation of redundant targets, while in the presence of two activated oncogenes, one often takes a dominant control of the target’s expression.

### Redundancy between the transcriptional profiles of oncogenic c-Myc, mutant KRAS and mutant p53 is a broad phenomenon in cancer cells

**Fig. 6:**
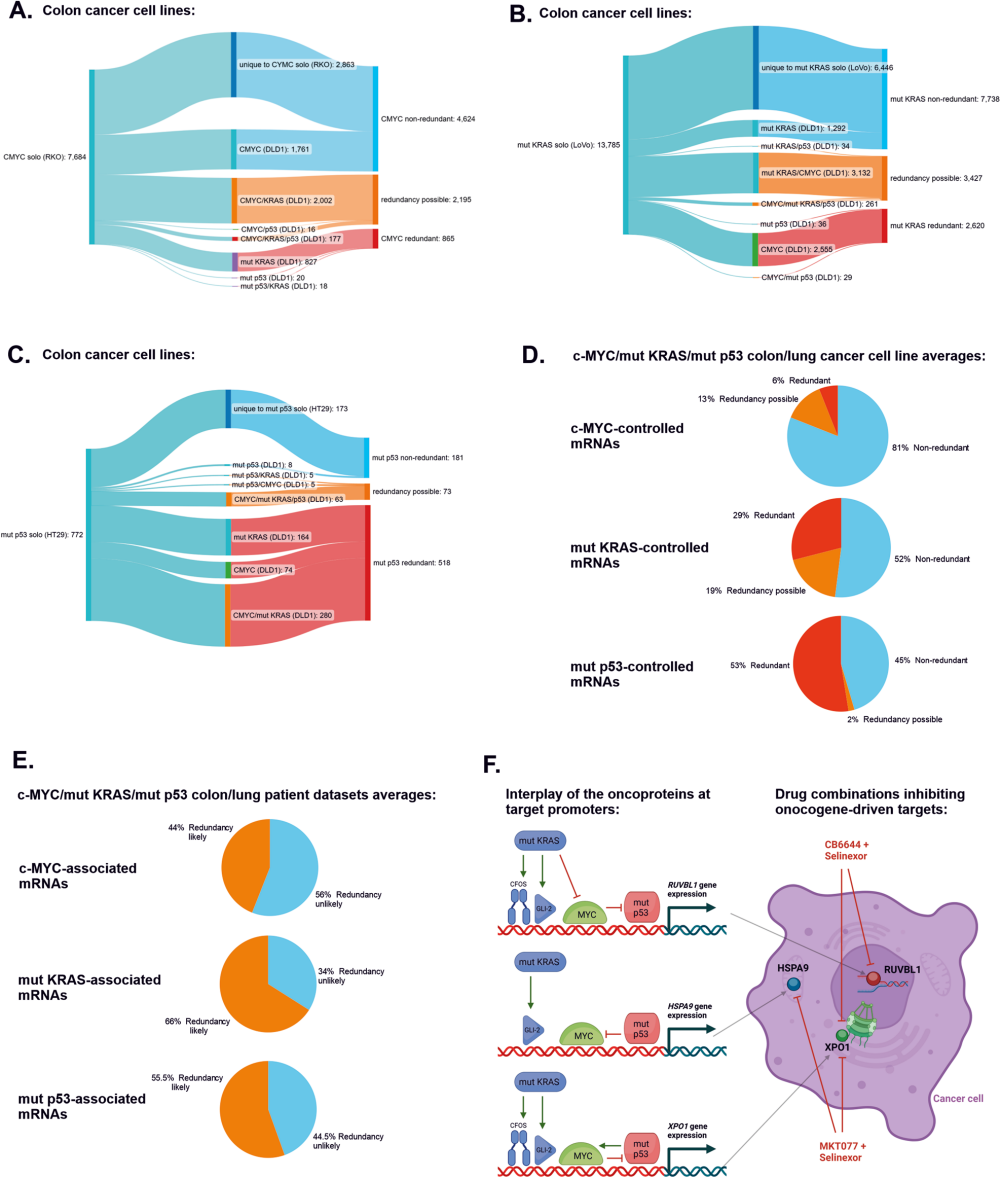
Redundancy in the transcriptional programs of oncogenic c-Myc, mutant KRAS, and mutant p53. Ribbon charts showing gene pools that are significantly dependent (FDR < 0.05; only coding mRNAs) on mutant *TP53*, mutant *KRAS*, or hyperactive *MYC* (**A**–**C**, respectively) in colon cancer cell lines with a single activated oncogene (left end), shared with three co-activated oncogenes (middle), resulting in: specificity of a gene pool to a single oncogene (non-redundant genes), sharing of a gene pool with co-expressed oncogenes (redundancy possible genes), or gene pools taken over by co-expressed oncogenes (redundant genes; right end). The data on the differentially expressed genes were derived from the CRISPR‒Cas9 experiment shown in Fig. 1A and Supplementary Table 2. **D** Percentages of non-redundant, possibly redundant, and redundant genes for each indicated oncogene, on average, in colon (**A**–**C**) and lung (Supplementary Fig. 6A–C) cancer cell lines. **E** Percentages of genes with likely and unlikely redundancies associated with the listed oncogenes, on average, in lung and colon cancer TCGA patient-derived expression datasets. The percentages were derived from the gene pool flow analyses shown in Supplementary Fig. 6D, E. **F** Scheme depicting modes of gene promoter control by the studied oncoproteins and targeting of the three proteins encoded by these genes - *RUVBL1*, *HSPA9*, *XPO1* - by drug combinations described in this study. The oncoproteins c-Myc, mutant KRAS, and mutant p53, dependent on the promoter, may activate one another (cooperation), bypass one another with parallel signaling to the promoter (redundancy) or inhibit one another (competition).

We investigated how broad is the phenomenon of redundancy in the transcriptional programs of oncogenic c-Myc, mutant KRAS, and mutant p53. First, we interrogated the transcriptional programs of the trio oncogenes determined earlier (Fig. 1, Supplementary Table 2). In the colon cancer cell lines, shown in Fig. 6A–C, the transcriptional program controlled by c-Myc was the most specific and the least redundant, as the majority of its target mRNAs were not co-controlled or taken over by mutant KRAS and p53 when co-expressed with c-Myc (Fig. 6A). However, in the case of mutant p53, the majority of transcripts controlled by the individually expressed oncogene were taken over by c-Myc and/or mutant KRAS in the background of co-expression of the three oncogenes (Fig. 6C). In the lung cancer cell lines c-Myc retained an even greater proportion of its solo-controlled mRNAs, while mutants of KRAS and p53 were strongly redundant with c-Myc (Supplementary Fig. 6A–C). On average, in the colon and lung cancer cell lines tested, the transcriptional program of c-Myc was the least redundant (6%), while the mutant p53 program was redundant in more than 50% of the cells in favor of mutant KRAS and/or c-Myc (Fig. 6D).

We validated these results against patient datasets from colon and lung cancers. This analysis was limited to associations in contrast to earlier direct manipulation of oncogenes; thus, we excluded combinations of three oncogenes from the analysis. Considering the average of results from colon and lung cancers the significantly altered mRNAs associated with the presence of c-Myc that were shared, and thus likely redundant, with other two oncogenes was 44%, while mutant p53 and mutant KRAS had 55.5 and 66% likely redundant genes, respectively (Fig. 6E).

These results indicate that the extent of oncogene redundancy is dependent on the oncogene and represents a phenomenon which may involve the majority of the oncogene’s transcription-driving potential.

## Discussion

Studies concerning the omics of oncogene co-activation in neoplastic cells have been carried out in most cases using two experimental systems. One involves patient material-centered studies, which allow to associate the activation of driver oncogenes with transcriptional/protein programs and phenotypic features and help define molecular subtypes of particular neoplasias [28–31]. Another experimental model involves the introduction or activation of oncogenes in a usually untransformed background in vitro or in vivo, followed by functional phenotypic and omics analyses [17, 32, 33]. Our study attempted to complement these methodologies with an approach involving CRISPR-mediated downregulation of activated oncogenes in transformed cells in vitro. This approach allowed us to directly compare the functional potential of each of the three major oncogenic drivers to determine the transcriptome and proteome in neoplastic cells addicted to the presence of the oncogenes. As expected from previous reports, we found broad overlaps in the pathways driven by each of the oncogenes, spanning all three analyzed cancer types. Considering the problems with direct targeting of the three studied oncogenes [5, 8, 12] and the universal nature of the identified overlaps, we found the potentially targetable three-protein signature of RUVBL1, HSPA9, and XPO1. Each of them individually was reported to be a promising target in cancer [34–36]; however, the synergistic pairwise drug combinations presented here exploit novel vulnerabilities against multiple cancer types.

Thus far, we expected to find cooperative control of the target genes by mutant p53, mutant KRAS, and c-Myc [2]. In contrast, the results presented here, obtained using in-house patient-derived samples and public patient datasets, suggested that the control of *RUVBL1*, *HSPA9*, and *XPO1* by the trio of the oncogenes was redundant rather than cooperative. We examined the underlying mechanism and found that each target gene is indeed controlled by each oncogene in parallel, with cooperation found only in the case of mutant p53 and *MYC* binding to the *XPO1* gene promoter. Moreover, we found that in most cases, a specific oncogene dominates the control over a particular target, “switching off” the influence of the co-activated oncogene. In the case of the *RUVBL1* gene, we observed a negative influence of mutant KRAS on the binding of c-Myc to the promoter, while c-Myc negatively influenced the binding of mutant p53 to the *HSPA9* promoter. This “oncogene competition” on target promoters has not been reported previously, while the existence of this mechanism has been suggested by rare studies on other competing oncogene functions – such as KRAS inactivating aspects of c-Myc signaling via MAPK pathway [37] or c-Myc suppressing mutant NRAS-driven immune response-inducing MHC class II expression [38]. Competition was also described between several transcription factors, including c-Myc, outside of the oncogenic context [39–41].

Since our mechanistic conclusions were based on a small, 3-gene signature, we attempted to assess the general prevalence of oncogene redundancy. In expression datasets from the studied cell lines we found that the proportion of genes whose control is carried over from one oncogene to two others depends on the oncogene – c-Myc being the most redundancy-resistant, and mutant p53 being redundant on average in more than 50% of its transcriptional program in lung and colon cell lines.

The concept of more and less “powerful” oncogenes, with mutant p53 largely dominated by the presence c-Myc/KRAS, could be explained by larger gene numbers and stronger expression changes induced by the latter (Fig. 1), leading to stochastic gene co-regulation. However, mutant KRAS significantly affected the expression of a greater number of mRNAs in colon cancer cells and was still more redundant with c-Myc than the other way around (Fig. 6A, B), suggesting that the mechanism is more complex than the simple domination of a broader expression regulator.

The scale of the redundancy, which reaches more than 50% of mutant p53-driven transcripts, is likely reflected in the controversy surrounding p53 mutants as driver oncoproteins. In the majority of studies, mutant p53 oncogenic gain-of-function has been found and explained [2, 42]. However, there are cancer models in which mutant p53 does not exhibit a clear gain-of-function; rather, it exerts only a dominant-negative effect and loss of its oncosuppressive function [43–46]. Such cases have been hypothesized to be dependent on the tissue/molecular context [42]. Most of these studies include models carrying mutant RAS family oncogenes [43, 44] or oncogenic c-Myc [46]. Based on the results shown here, we propose that other activated oncogenes are important components of the background context, limiting the range of influence of mutant p53 on cancer cells. Our results also suggest that a similar interplay, involving redundancy and competition, could happen between mutant p53 and other oncogenic regulators.

The extent to which the “race for power” between oncogenes involves oncogene competition and includes drivers other than the trio described here remains to be evaluated. Nevertheless, extensive oncogene redundancy provides a warning of possible resistance arising from direct targeting of driver oncoproteins, as already reported in clinical trials of FGFR inhibitors [47] or pre-clinical pan-RAS inhibition compensated by c-Myc-driven transcription [10]. This mandates a search for targetable signatures, such as RUVBL1, HSPA9, and XPO1, whose redundancy is limited by directly non-overlapping roles in cancer cell metabolism.

### Supplementary information

Supplementary Figures and Legends

Supplementary Table 1

Supplementary Table 2

Supplementary Table 3

Supplementary Table 4

Supplementary Table 5

Supplementary Table 6

Supplementary Table 7

Uncropeed Western Blots

## Data Availability

Raw proteomics and transcriptomics results are available in respective databases under following accession numbers: GSE239817 (GEO database; RNA-seq), PXD037398 (PRIDE database; proteomics). The analyzed omics results, pathway association, IC50/viability and drug concentration detailed data are available in Supplementary Tables 1–5, as described in the Results section.
