# Supplementary material for: A common druggable signature of oncogenic c-Myc, mutant KRAS and mutant p53 reveals functional redundancy and competition among oncogenes in cancer
Source: Cell Death Dis. 2024 Aug 31;15(8):638. doi: 10.1038/s41419-024-06965-3 (PMC11365971; doi:10.1038/s41419-024-06965-3)
Supplement: Supplementary file 1 — Supplementary Figures and Legends [file 41419_2024_6965_MOESM1_ESM.pdf]

Supplementary Figure 1

A.

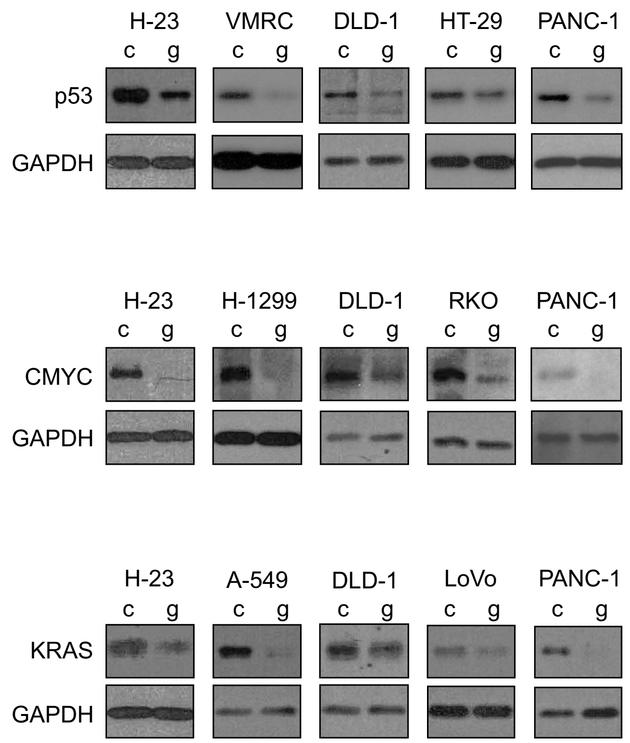

B.

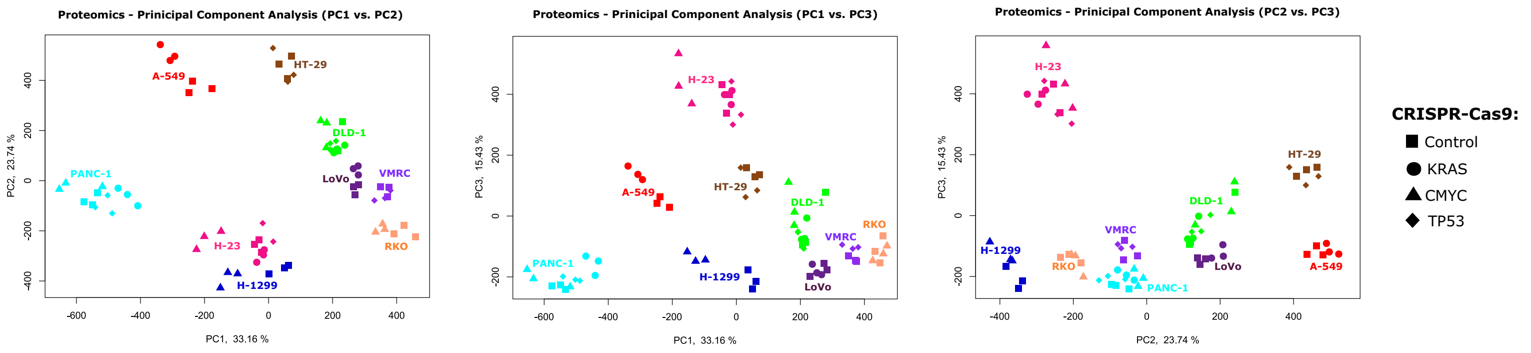

C.

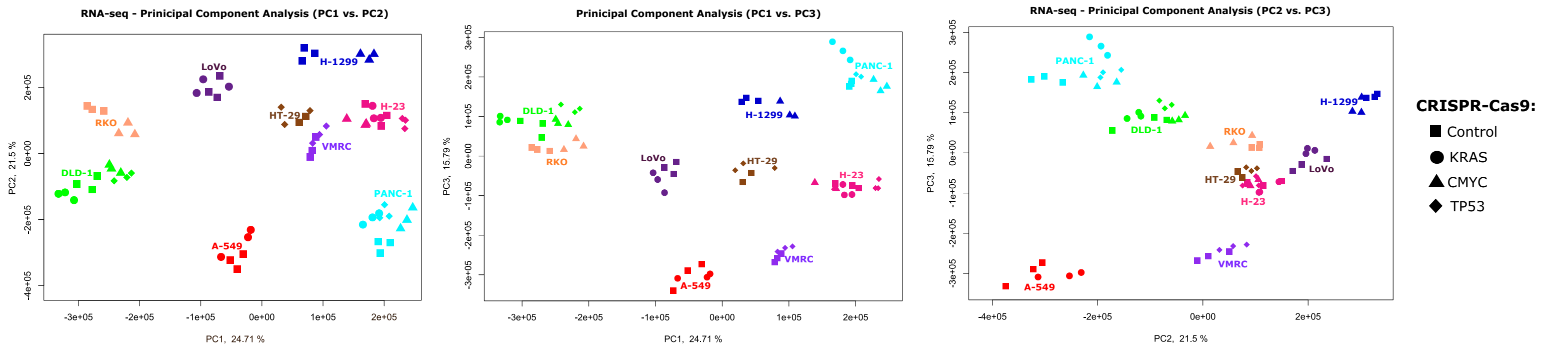

Supplementary Figure 1 **A.** Western blots showing representative samples of control and oncogene-targeting gRNA in CRISPR-Cas9-mediated downregulation of indicated oncoproteins, whose large-scale analysis is shown in Fig. 1. Analyzed cell line is indicated above each WB. **B.** Principal component analysis (PCA) of proteomics samples described in Fig. 1. Cell lines are marked by colors and names, while gRNAs used in CRISPR-Cas9 experiments are indicated by symbol shapes. **C.** PCA for mRNAs resulting from RNA-sequencing described in Fig. 1 done and shown as in (B).

Supplementary Figure 2

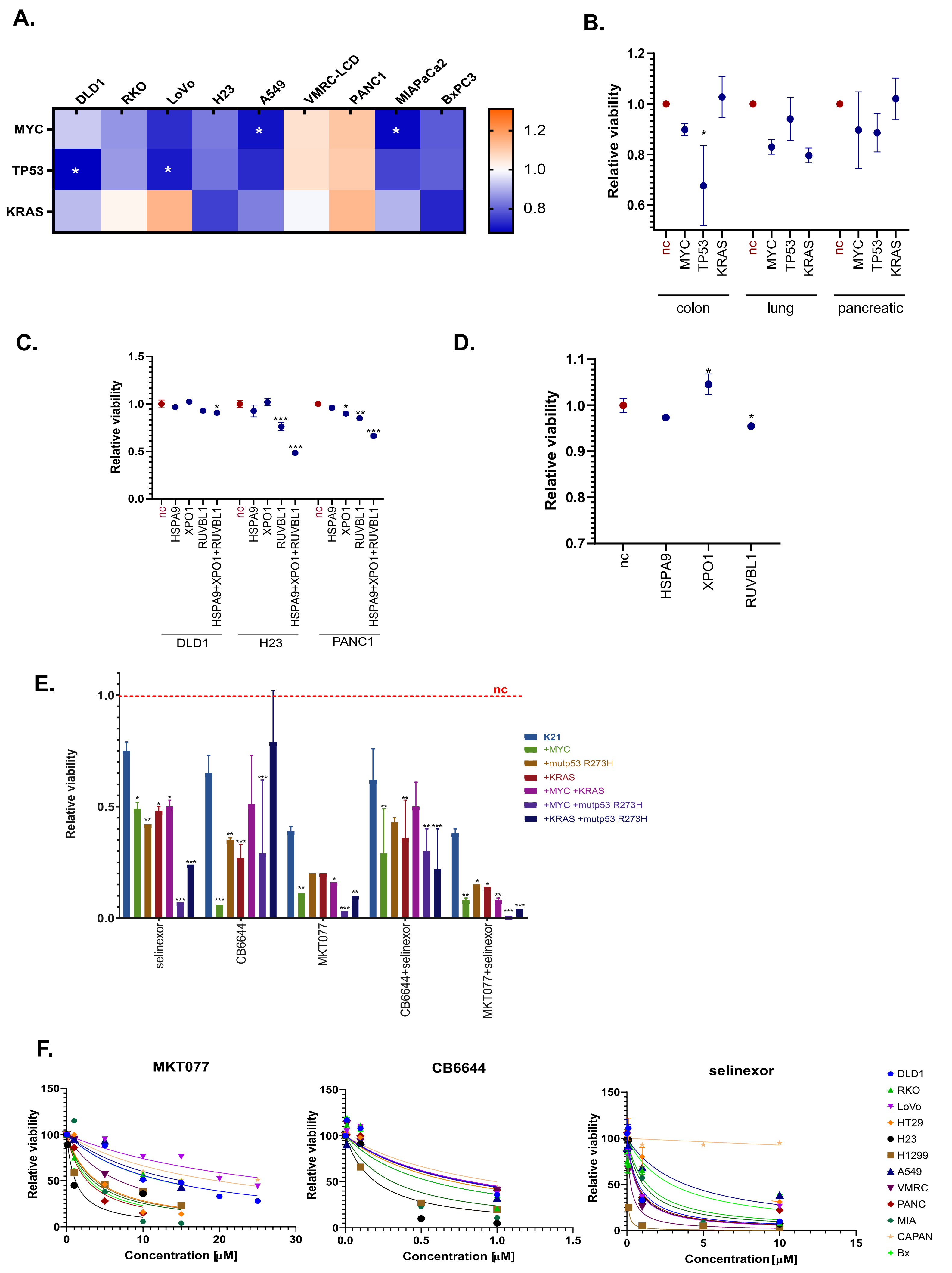

Supplementary Figure 2 **A.** Viability of colon, lung, and pancreatic cancer cell lines transfected with siRNAs targeting indicated oncogenes. Resazurin assay was used for viability measurement 48h post siRNA transfection. Data in the heatmap are presented as means of 2 replicates for each cell line and analyzed with Two-way ANOVA (uncorrected Fisher's LSD) versus siRNA negative control. **B.** Data from (A) grouped in oncogene-wise manner within given cancer type, presenting the average impact of oncogene silencing on viability of cancer cell lines harboring alteration in given oncogene. Results are a mean of n=2 (*TP53* in colon, *MYC* in lung), n=6 (*TP53* in pancreatic) or n=4 (the rest of results), error bars represent SEM. Data were analyzed as in (A). **C.** The effectiveness of simultaneous silencing of targets representing different molecular pathways (RUVBL1, XPO1, and HSPA9) versus siRNA negative control conducted in DLD1, H23, and PANC1 cell lines. Viability was measured as in (A). Data are presented and analyzed as in (B). **D.** The impact of RUVBL1, XPO1, and HSPA9 silencing with the use of siRNA on normal cell lines (fibroblast F02 and F03). Results are a mean of n=4, error bars represent SEM. Data were analyzed with One-way ANOVA (uncorrected Fisher's LSD) versus siRNA negative control. **E.** Effectiveness of selected inhibitor combinations depending on the oncogene background. MKT077 with selinexor and CB6644 with selinexor were tested in K21 fibroblast overexpressing oncogenes (single or in pairs). Viability measurement was performed 72h post transfection using ATPlite reagent. Each result is a mean of two replicates, error bars represent SEM. Data were analyzed with Two-way ANOVA (uncorrected Fisher's LSD). (A)-(E): *p < 0,05, **p<0,01, ***p<0,001. **F.** Titration of indicated inhibitor concentrations in listed cancer cell lines shown as function of their viability measured 72h post treatment with the use of ATPlite. The fitted inhibition curves were used to calculate IC50 values listed in Supplementary Table 5.

Supplementary Figure 3

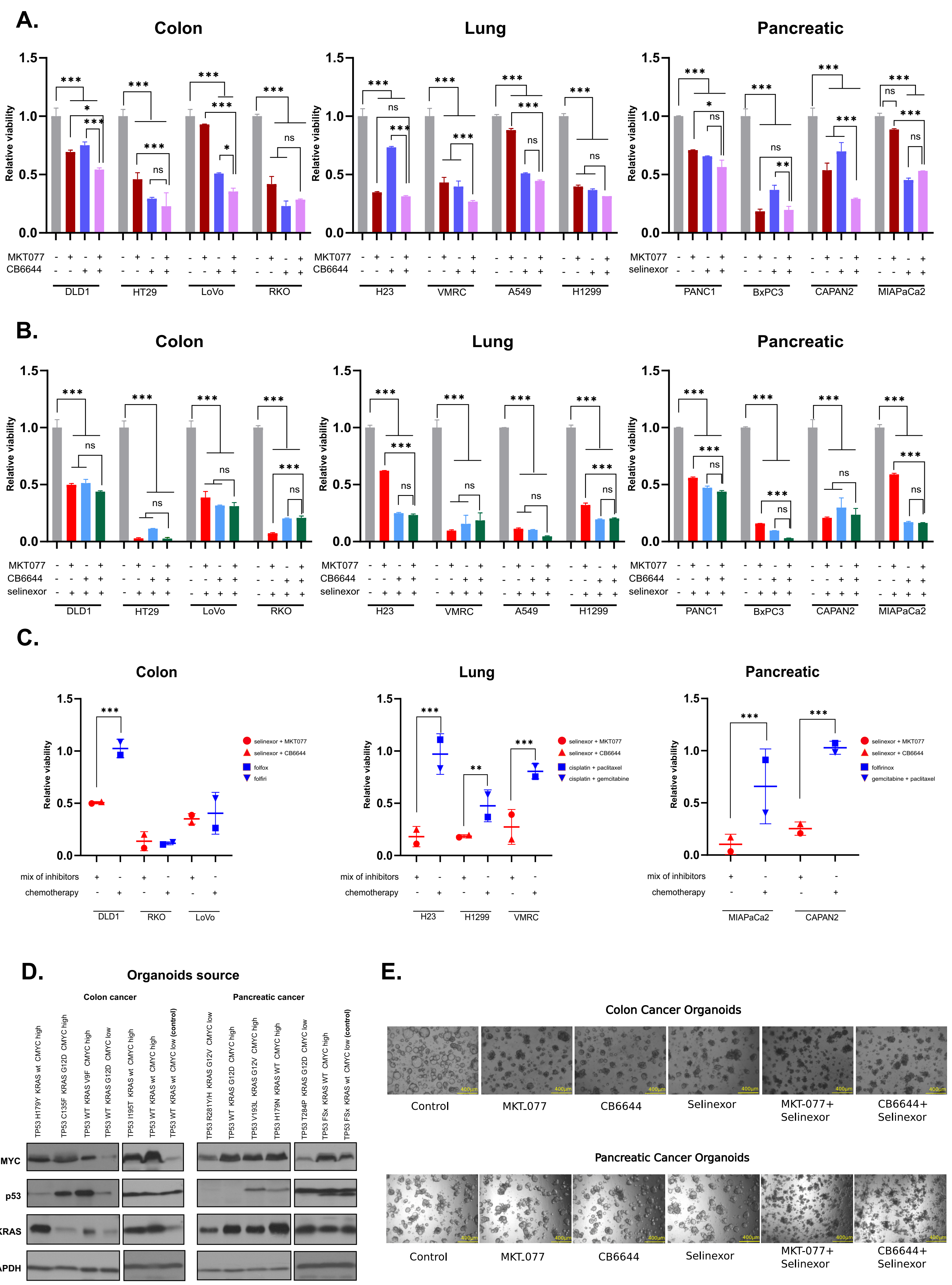

Supplementary Figure 3 **A.** Viability of colon, lung and pancreatic cancer cell lines treated with CB6644, MKT077, and combination of both inhibitors. **B.** Impact of MKT077, CB6644, and selinexor mixture on viability of colon, lung, and pancreatic cancer cell lines. Viability in (A-B) was measured with ATPlite reagent, 72h post treatment with drug concentrations calculated individually for each cell line (based on IC50 values in Supplementary Table 5). Each bar represents mean of two replicates with SD. Data were analyzed with two-way ANOVA with Tukey's correction, *p < 0,05, ***p<0,01. **C.** Comparison of viability of indicated cell lines with most effective inhibitor combinations introduced in this study (selinexor+MKT077 and selinexor+CB6644) with two listed standard chemotherapeutic protocols for each cancer type (means of the viability results with SEM is shown). Data were analyzed with one-way ANOVA with Dunnett correction, **p<0,01, ***p<0,001. **D.** Western blot indicating p53, KRAS, and c-Myc protein levels in the organoid cultures used in Fig. 3D, E, with the listed status of the three oncogenes in each culture. **E.** Phase-contrast microscopy of colon and pancreatic cancer organoids derived from tumor patient's tissues. Representative pictures of the organoid culture morphology post treatment with MKT-077, CB6644, selinexor, and combinations of inhibitors for viability test shown in Fig. 3 D, E. Scale bar is 400µm.

Supplementary Figure 4

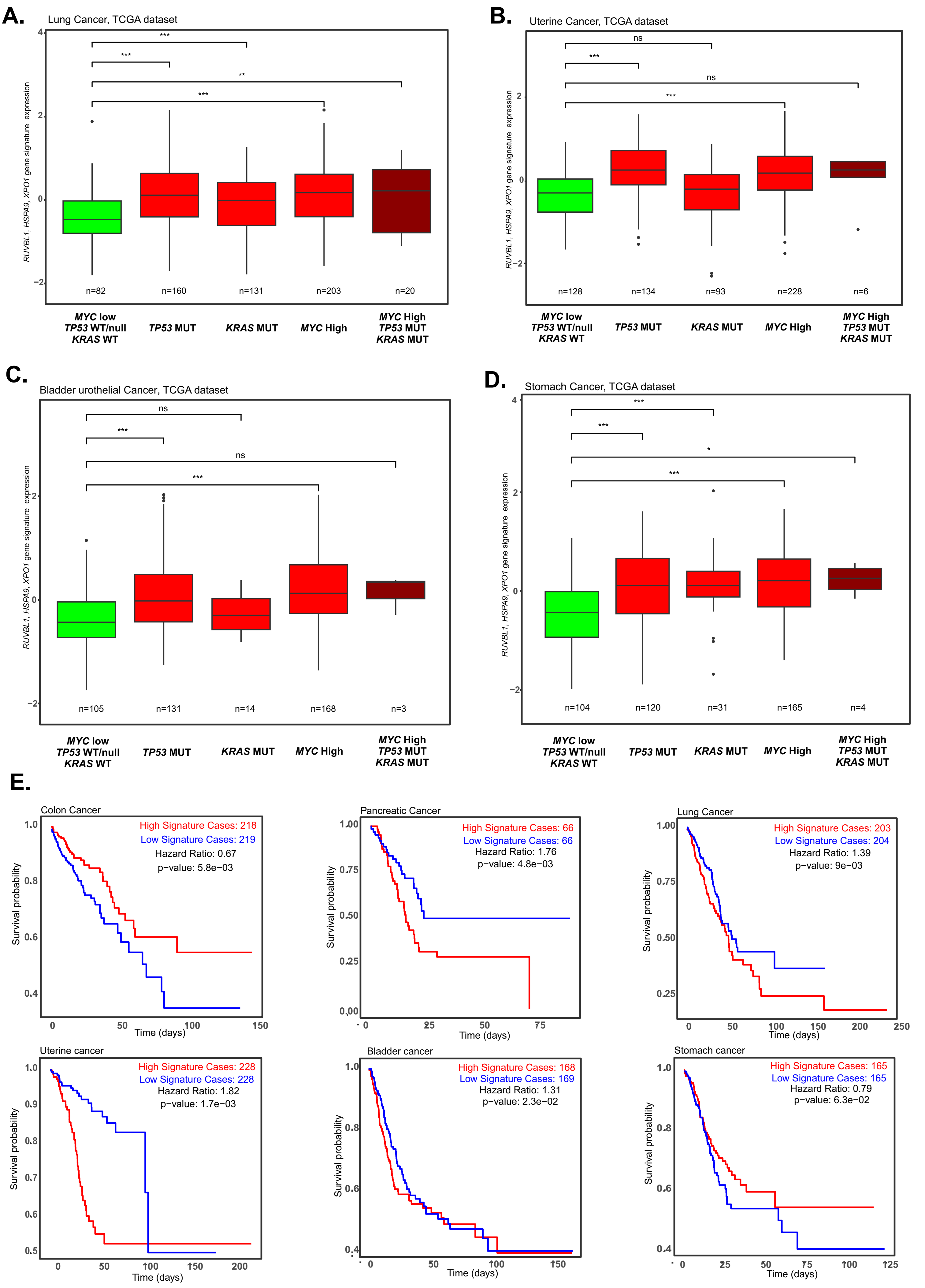

F.

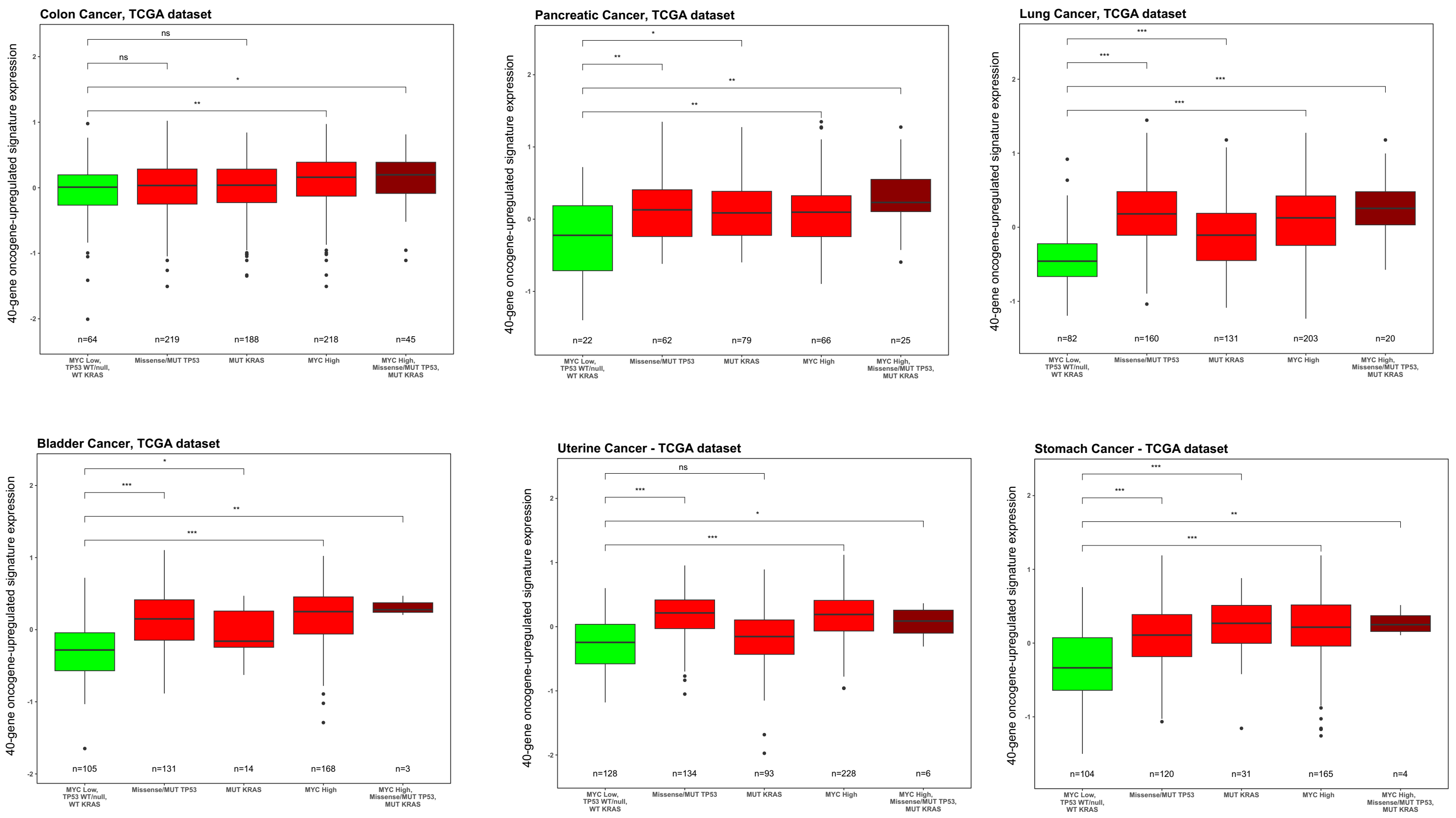
Colon Cancer, TCGA dataset
Pancreatic Cancer, TCGA dataset
Lung Cancer, TCGA dataset
Bladder Cancer, TCGA dataset
Uterine Cancer - TCGA dataset
Stomach Cancer - TCGA dataset
40-gene oncogene-upregulated signature expression
MYC Low, TP53 WT/null, WT KRAS
Missense/MUT TP53
MUT KRAS
MYC High
MYC High, Missense/MUT TP53, MUT KRAS
n=64
n=219
n=188
n=218
n=45
n=22
n=62
n=79
n=66
n=25
n=82
n=160
n=131
n=203
n=20
n=105
n=131
n=14
n=168
n=3
n=128
n=134
n=93
n=228
n=6
n=104
n=120
n=31
n=165
n=4

G. Figure 4 colon cancer samples:

Patient:	Tumor tissue:	Diagnosis:	Oncogenes:
CC 1	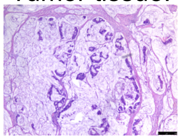	Mucinous<br>cecal<br>adenocarcinoma<br>pT4a, N2a,<br>M1	TP53 R273H<br>KRAS WT<br>MYC low
CC 2	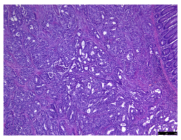	Colon<br>adenocarcinoma<br>G2<br>pT4a, N2b<br>M1a	TP53 WT<br>KRAS WT<br>MYC high
CC 3	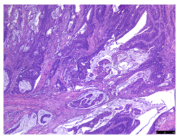	Colon<br>adenocarcinoma<br>G2<br>pT2, N1b<br>R0	TP53 WT<br>KRAS WT<br>MYC high
CC 4	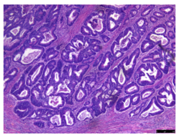	Colon<br>adenocarcinoma<br>G2<br>pT3, N0<br>R0	TP53 WT<br>KRAS WT<br>MYC low
CC 5	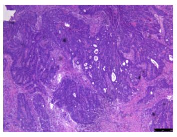	Adenocarcinoma of the<br>rectosigmoid flexure<br>G2<br>pT2, N1a<br>R0	TP53 212STOP<br>KRAS WT<br>MYC low
CC 6	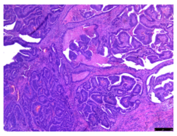	Cecal<br>adenocarcinoma<br>G2<br>pT2, N1b<br>R0	TP53 WT<br>KRAS WT<br>MYC high
CC7	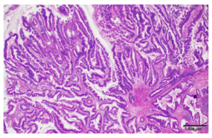	Colon<br>adenocarcinoma<br>G1<br>pT3, N0<br>R0	TP53 WT<br>KRAS WT<br>MYC high
CC8	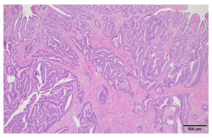	Colon<br>adenocarcinoma<br>G1<br>pT2, N0<br>R0	TP53 R282W<br>KRAS G12C<br>MYC low
CC9	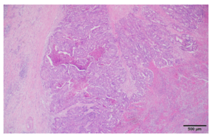	Colon<br>adenocarcinoma<br>G2<br>pT3, N0<br>R0	TP53 WT<br>KRAS WT<br>MYC high
CC10	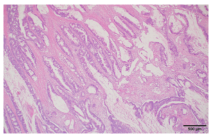	Colon<br>adenocarcinoma<br>G2<br>pT3, N0<br>R0	TP53 WT<br>KRAS WT<br>CMYC high
CC11	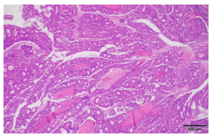	Colon<br>adenocarcinoma<br>G2<br>pT3, N1b<br>R0	TP53 WT<br>KRAS G12D<br>MYC low
CC12	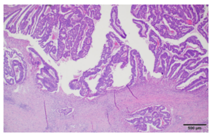	Colon<br>adenocarcinoma<br>G2<br>pT3, N0<br>R0	TP53 N239S<br>KRAS G12D<br>MYC low
CC13	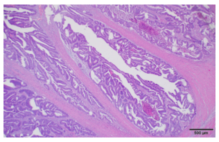	Colon<br>adenocarcinoma<br>G2<br>pT3, N1a<br>R0	TP53 WT<br>KRAS WT<br>MYC high
CC14	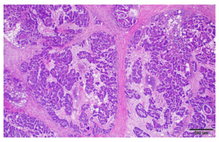	Colon<br>adenocarcinoma<br>G3<br>pT4a, N2b<br>R0	TP53 WT<br>KRAS WT<br>MYC low
CC15	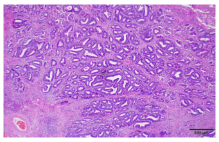	Colon<br>adenocarcinoma<br>G2<br>pT3, N0<br>R0	TP53 WT<br>KRAS WT<br>MYC high
CC16	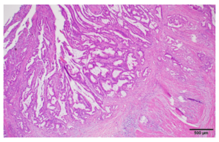	Colon<br>adenocarcinoma<br>G2<br>pT3, N2a<br>R0	TP53 R248Q<br>KRAS WT<br>MYC low
CC17	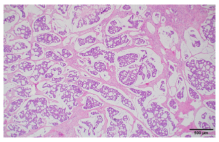	Colon<br>adenocarcinoma<br>G2<br>pT3, N0<br>R0	TP53 WT<br>KRAS WT<br>MYC high
CC18	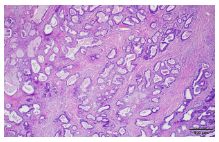	Colon<br>adenocarcinoma<br>G2<br>pT3, N0<br>R0	TP53 WT<br>KRAS WT<br>MYC high
CC19	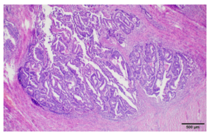	Colon<br>adenocarcinoma<br>G2<br>pT2, N0<br>R0	TP53 R258K<br>KRAS G12D<br>MYC low
CC20	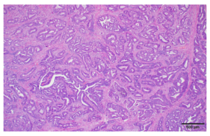	Colon<br>adenocarcinoma<br>G2<br>pT2, N0<br>R0	TP53 R273H<br>KRAS WT<br>MYC high
CC21	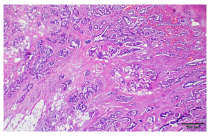	Colon<br>adenocarcinoma<br>G2<br>pT3, N0<br>R0	TP53 WT<br>KRAS WT<br>MYC low
CC22	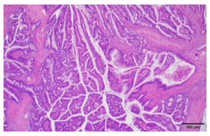	Colon<br>adenocarcinoma<br>G2<br>pT3, N0<br>R0	TP53 WT<br>KRAS G13D<br>MYC low
CC23	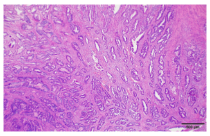	Colon<br>adenocarcinoma<br>G2<br>pT3, N1b<br>M1	TP53 R175H<br>KRAS WT<br>MYC low
CC24	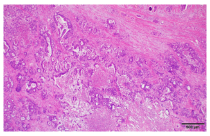	Colon<br>adenocarcinoma<br>G2/G3<br>pT4a, N1b<br>M1	TP53 WT<br>KRAS WT<br>MYC low

H. Figure 3 organoid colon cancer samples:

Patient:	Tumor tissue:	Diagnosis:	Oncogenes:
CC25	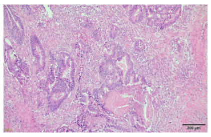	Rectal<br>adenocarcinoma<br>G2<br>pT3, N0<br>R0	TP53 H179Y<br>KRAS WT<br>MYC high
CC26	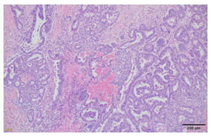	Colon<br>adenocarcinoma<br>G2<br>pT4a, N0<br>R0	TP53 C135F<br>KRAS G12D<br>MYC high
CC27	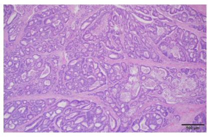	Colon<br>adenocarcinoma<br>G2<br>pT3, N0<br>N0	TP53 WT<br>KRAS V9F<br>MYC high
CC28	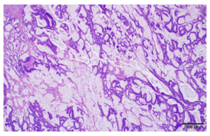	Colon<br>adenocarcinoma<br>Gx<br>pT3, N2b<br>R0	TP53 WT<br>KRAS G12D<br>MYC low

I. Figure 4 pancreatic cancer samples:

Sample ID:	Tumor tissue:	Diagnosis:	Oncogenes:
PDA1	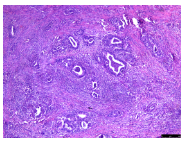	Pancreatic adenocarcinoma<br>G3<br>pT2, N1<br>R0	TP53 WT<br>KRAS WT<br>MYC low
PDA2	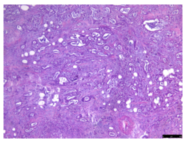	Pancreatic adenocarcinoma<br>G2<br>pT2, N2<br>R1	TP53 WT<br>KRAS WT<br>MYC low
PDA3	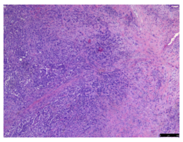	Pancreatic adenocarcinoma<br>G3<br>pT2, N0<br>R0	TP53 C238F<br>KRAS WT<br>MYC high
PDA4	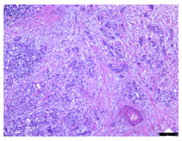	Pancreatic adenocarcinoma<br>G3<br>pT3, N1<br>R1	TP53 WT<br>KRAS G12R<br>MYC low
PDA5	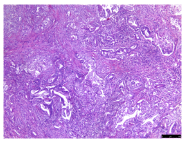	Pancreatic adenocarcinoma<br>G2<br>pT3, N2<br>R1	TP53 WT<br>KRAS G12D<br>MYC low
PDA6	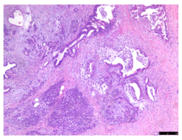	Pancreatic adenocarcinoma<br>G2<br>pT3, N0<br>R1	TP53 WT<br>KRAS WT<br>MYC low
PDA7	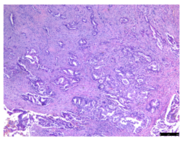	Pancreatic adenocarcinoma<br>G2<br>pT2, N1<br>R1	TP53 WT<br>KRAS WT<br>MYC high
PDA8	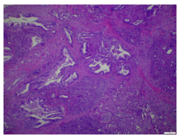	Pancreatic adenocarcinoma<br>G2<br>pT2, N2<br>R1	TP53 WT<br>KRAS G12D<br>MYC low
PDA9	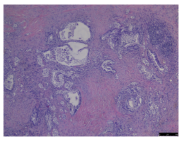	Pancreatic adenocarcinoma<br>G2<br>pT3, N1<br>R1	TP53 WT<br>KRAS WT<br>MYC high
PDA10	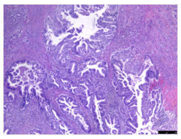	Pancreatic adenocarcinoma<br>G2<br>pT3, N1<br>R1	TP53 WT<br>KRAS WT<br>MYC low
PDA11	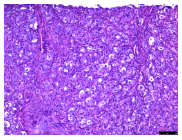	Pancreatic adenocarcinoma<br><br>Liver metastsis	TP53 R193D<br>KRAS G12D<br>MYC low
PDA12	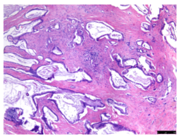	Pancreatic adenocarcinoma<br>Gx<br>pT1c, N1, M1<br>R0	TP53 WT<br>KRAS WT<br>MYC low
PDA13	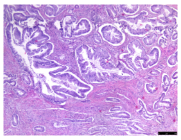	Pancreatic adenocarcinoma<br>G1<br>pT1, N1<br>R1	TP53 WT<br>KRAS WT<br>MYC low
PDA14	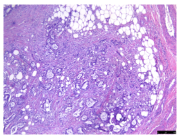	Pancreatic adenocarcinoma<br>G1<br>pT2, N0<br>R1	TP53 WT<br>KRAS WT<br>MYC high

J. Figure 3 pancreatic cancer samples:

Sample ID:	Tumor tissue:	Diagnosis:	Oncogenes:
PDA15	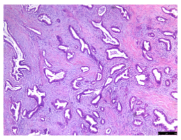	Pancreatic adenocarcinoma<br>G2<br>pT3, N0<br>R1	TP53 D281Y/H<br>KRAS WT<br>MYC low
PDA16	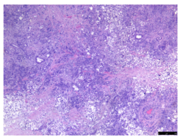	Pancreatic adenocarcinoma<br>G3<br>pT2, N0<br>R0	TP53 WT<br>KRAS G12D<br>MYC high
PDA17	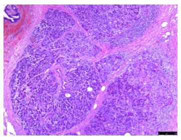	Pancreatic adenocarcinoma<br>G3<br>pT3b, N1<br>R1	TP53 H179N<br>KRAS WT<br>MYC high
PDA18	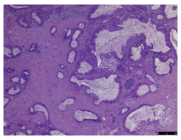	Pancreatic adenocarcinoma<br>G2<br>pT2, N1<br>R1	TP53 V193L<br>KRAS G12V<br>MYC high

Supplementary Figure 4 A. Comparative expression analysis of a 3-gene signature consisting of *RUVBL1, HSPA9,* and *XPO1* in TCGA-derived patient samples of lung cancer (mean value of three genes in each patient was used to calculate the sample distribution in the box plot), stratified according to the listed *TP53, KRAS* (only point mutations), and *MYC* expression status. The sample was included in "MYC high" if the *MYC* expression was above the *MYC* average expression level for all the patients in the graph. Student's t-test was used to obtain the indicated p-values. **B-D.** As in (A) for TCGA-derived patient samples of uterine, bladder urothelial, and stomach cancers, respectively. **E.** Kaplan-Meier survival analysis of patients with the indicated cancer types, stratified according to the mean expression level of the *RUVBL1, HSPA9,* and *XPO1* signature ("low" and "high"). Log rank test was used to obtain shown p-values for the difference between high and low expression levels of the signature. **F.** Expression analysis as in (A)-(D) for a 40-gene signature, derived from 56 genes listed in Fig. 1F which on average had downregulated mRNA expression levels across all cell line-derived datasets with downregulated oncogenes (genes expected to be activated by the oncoproteins). **G.** Hematoxylin and eosin staining histopathology photos, patient information/diagnosis and oncogene mutation/level status for *TP53*, *KRAS,* and *MYC* for colon cancer samples used in Fig. 4A. **H.** As in (G) for colon cancer samples used to establish organoid cultures tested in Fig. 3D. **I.** Hematoxylin and eosin staining histopathology photos, patient information/diagnosis and oncogene mutation/level status for *TP53*, *KRAS,* and *MYC* for pancreatic cancer samples used in Fig. 4B. **J.** As in (I) for pancreatic cancer samples used to establish organoid cultures tested in Fig. 3E.

For (A)-(D) and (F) n-numbers are given for each condition at the x-axis and significance thresholds are: $^*p<0.05$, $^{**}p<0.01$, $^{***}p<0.001$.

Supplementary Figure 5

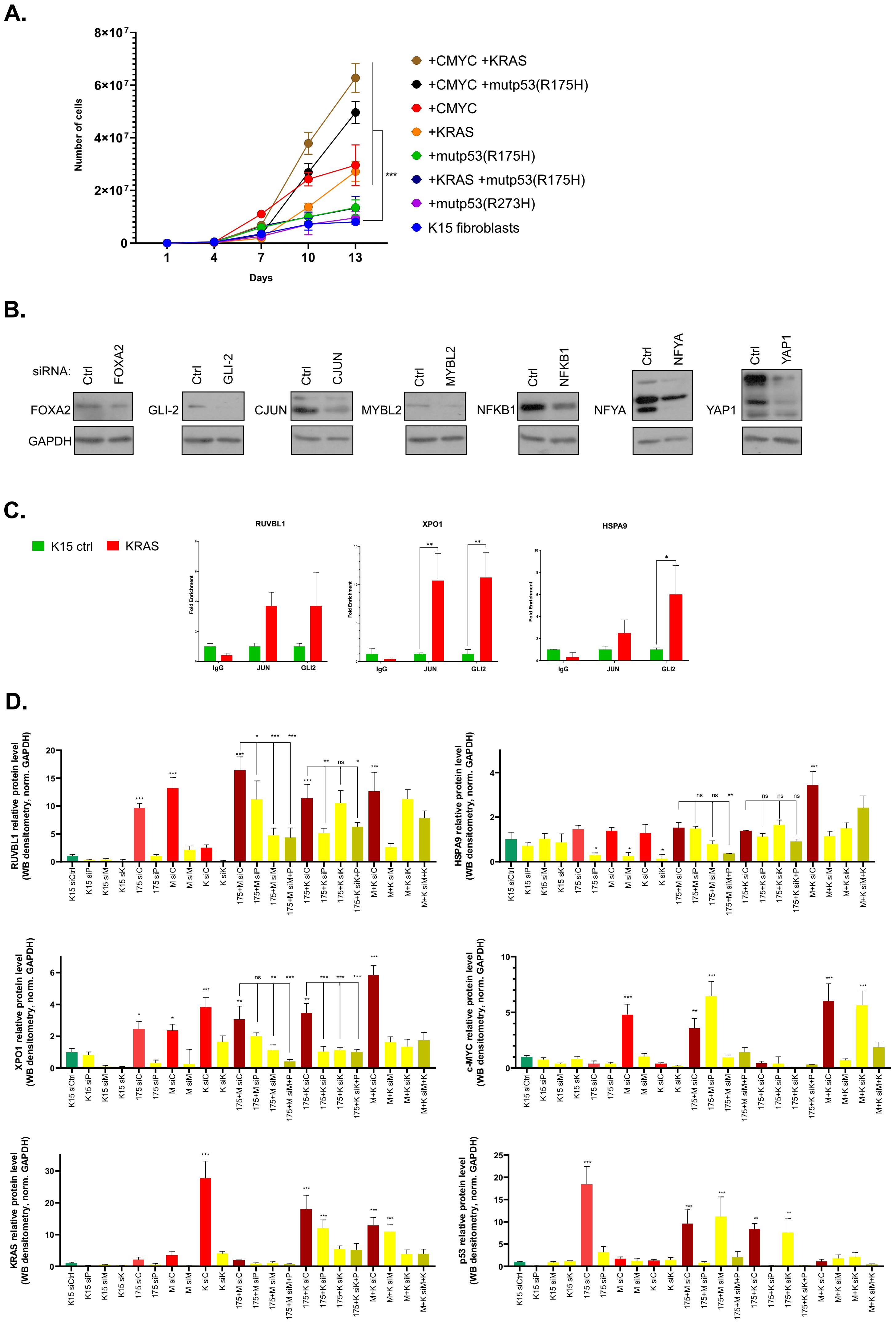

Supplementary Figure 5 A. Growth of K15 human fibroblasts with introduced listed single or double oncogenes. Cells were counted in 4-3 days intervals as shown in the graph. Means of n=2 are shown with SD, two-way ANOVA test with Dunnett correction, ***p<0.001. **B.** Western blots showing samples of K15 fibroblast cells with siRNA mediated silencing of indicated candidate co-factors of mutant KRAS vs. non-specific silencing controls, related to Fig. 5G. Detected protein is indicated next to each blot with GAPDH detection as housekeeping control in all cases. **C.** Chromatin immunoprecipitation-derived qPCR result of the promoter regions in the indicated genes, performed in either control K15 immortalized human fibroblasts or K15 fibroblasts with mutant KRAS stable overexpression. The PCR results were normalized to the levels of DNA precipitated in control K15 cells by each listed used antibody. **D.** Densitometric analysis was performed in samples shown in Fig. 5D (for p53 with its own GAPDH control shown in uncropped membranes figure) and in another biological replicate (not shown) to obtain average normalized values, relative to GAPDH, for each protein shown in Fig. 5D. One-way ANOVA with Dunnett correction was used to compare all samples with the control cells (K15 siCtrl, average normalized to 1) and - as indicated - with selected double oncogene overexpressions; overexpression and siRNAs are marked as described for Fig. 5A-C; n=2, *p<0.05, **p<0.01, ***p<0.001.

Supplementary Figure 6

A. **Lung cancer cell lines:**

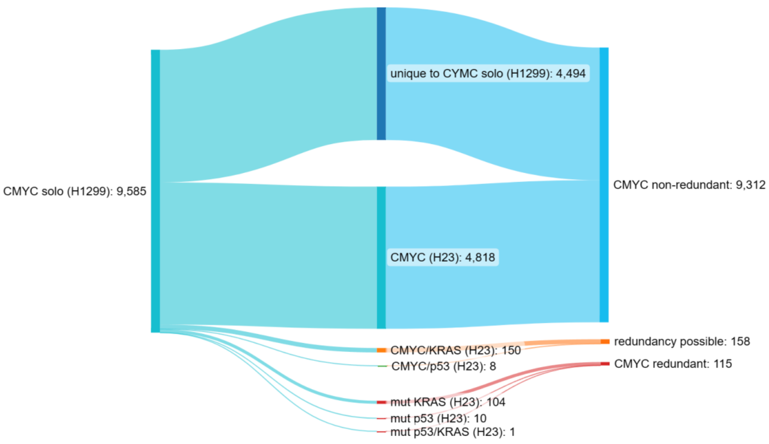

B. **Lung cancer cell lines:**

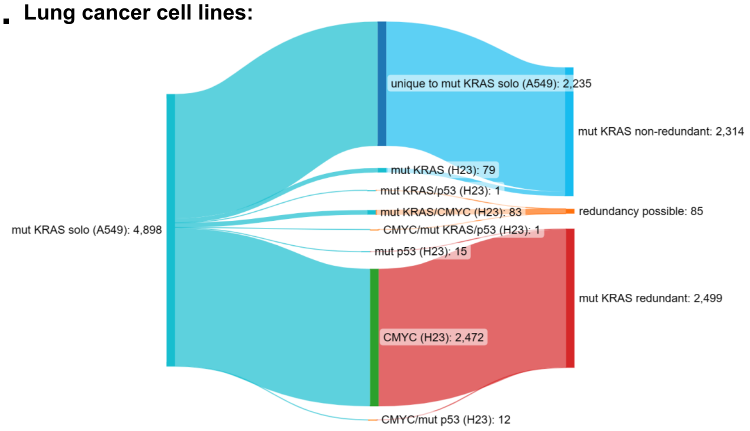

C. **Lung cancer cell lines:**

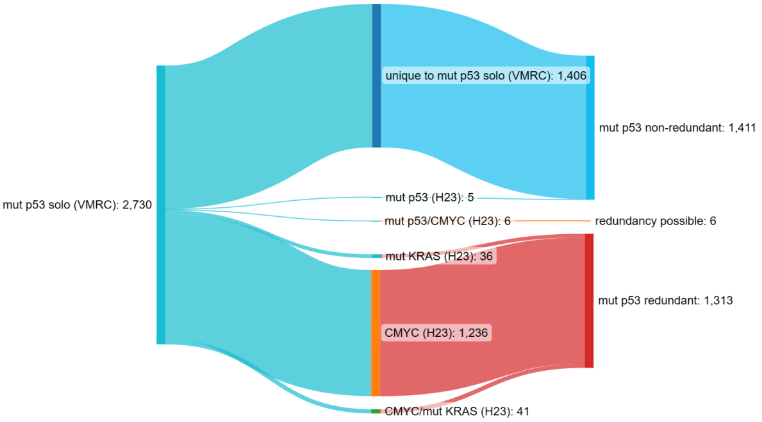

D. **Colon cancer patient datasets:**

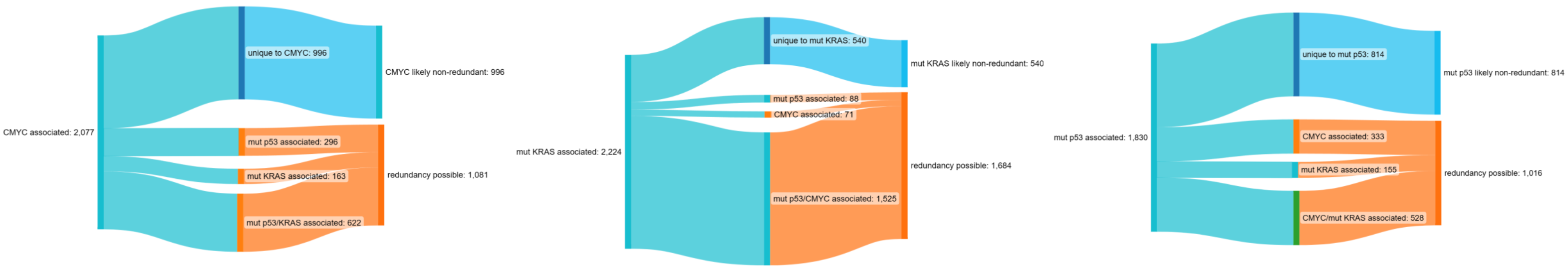

E. **Lung cancer patient datasets:**

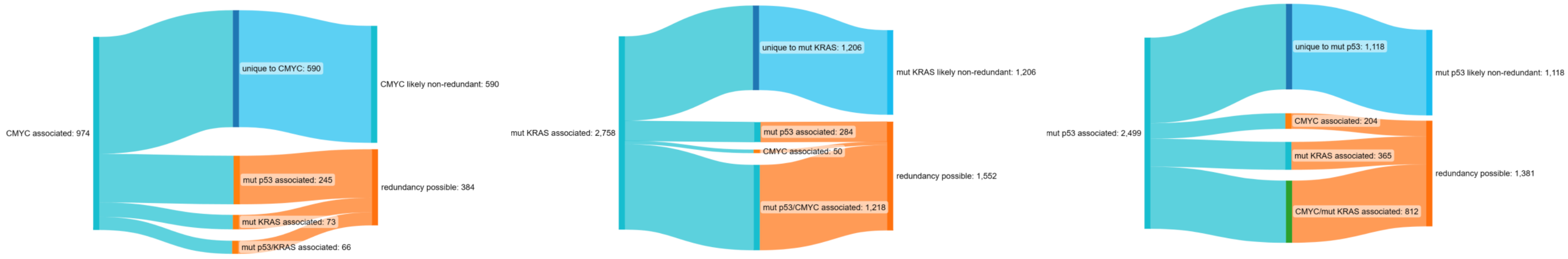

Supplementary Figure 6 A-C. Ribbon charts showing gene pools dependent significantly (FDR<0.05; only coding mRNAs) on mutant *TP53*, mutant *KRAS,* or hyperactive *MYC* (respectively) in lung cancer cell lines with single activated oncogenes (left end) shared with cell line with three co-activated oncogenes (middle), resulting in a specificity to the single oncogene (non-redundant genes), sharing with the co-expressed oncogenes (redundancy possible genes), or take-over by the co-expressed oncogenes from the program of the single oncogene (redundant genes; right end). The data on differentially expressed genes is derived from the CRISPR-Cas9 experiment in Fig. 1A and Supplementary Table 2. **D.** Ribbon charts showing gene pools associated significantly (FDR<0.05; only coding mRNAs) to the presence of mutant *TP53*, mutant *KRAS,* or hyperactive *MYC* (left end in consecutive graphs) in TCGA-derived colon cancer patient dataset, shared or not with other co-activated oncogenes (middle), resulting in a specificity to the single oncogene (likely non-redundant genes), or sharing with the co-expressed oncogenes (redundancy possible genes). **E.** As in (D) for TCGA-derived lung cancer patient dataset. Data from (D)-(E) is summarized in Fig. 6E.
