# Supplementary material for: A common druggable signature of oncogenic c-Myc, mutant KRAS and mutant p53 reveals functional redundancy and competition among oncogenes in cancer
Source: Cell Death Dis. 2024 Aug 31;15(8):638. doi: 10.1038/s41419-024-06965-3 (PMC11365971; doi:10.1038/s41419-024-06965-3)
Supplement: Supplementary file 9 — Uncropeed Western Blots [file 41419_2024_6965_MOESM9_ESM.pdf]

Supplementary Figure 7 - uncropped western blot photos realted to Fig. 5

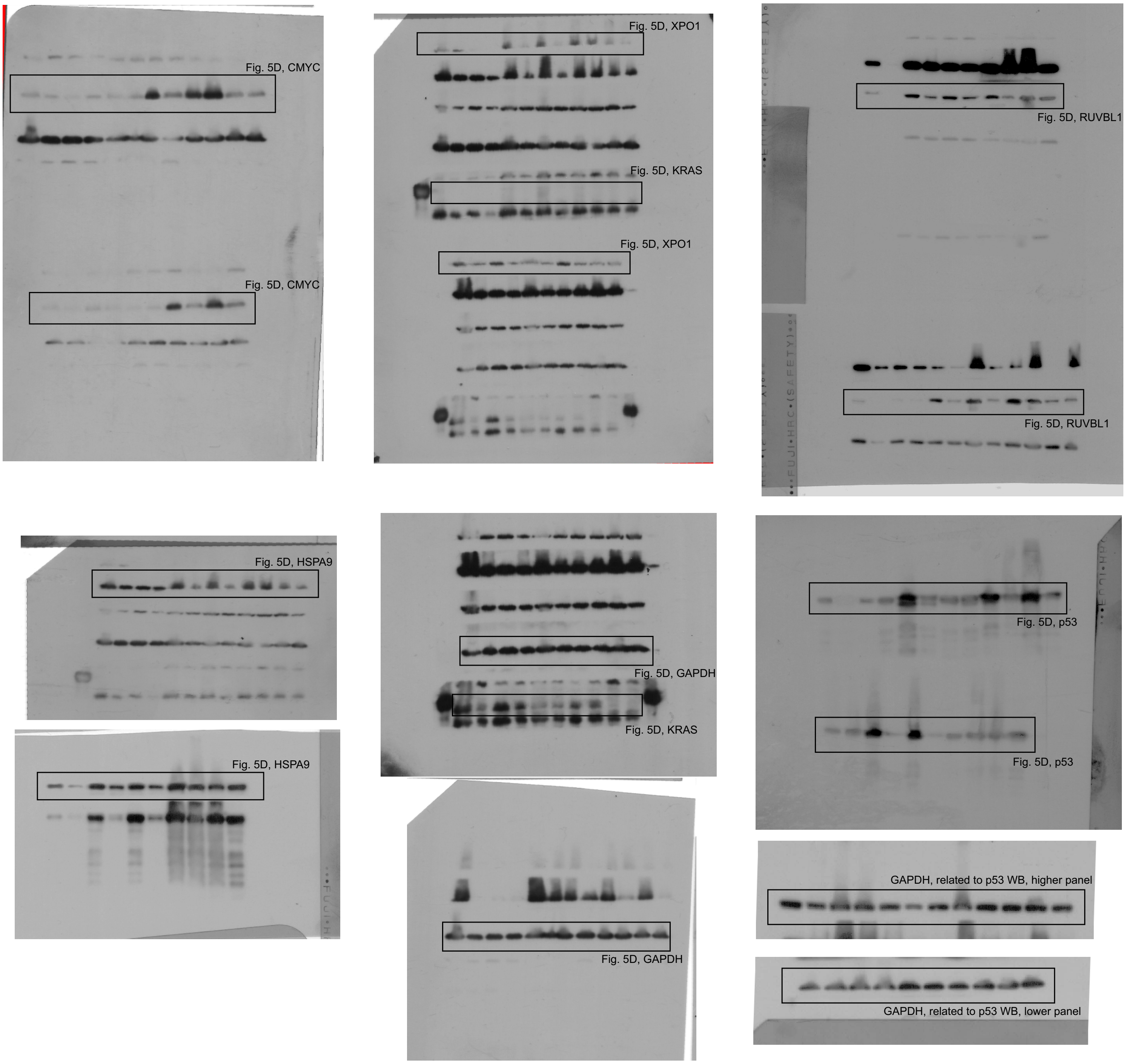

Supplementary Figure 7 – Uncropped western blots related to Figure 5D. Areas shown in the figure are indicated by black frames.
